# Comparison of the Effects of Potassium Sulphate and Potassium Chloride Fertilisation on Quality Parameters, Including Volatile Compounds, of Potato Tubers After Harvest and Storage

**DOI:** 10.3389/fpls.2022.920212

**Published:** 2022-07-11

**Authors:** Lisanne Wilmer, Elke Pawelzik, Marcel Naumann

**Affiliations:** Department of Crop Sciences, Division Quality of Plant Products, University of Göttingen, Göttingen, Germany

**Keywords:** potato, quality, potassium, K_2_SO_4_, KCl, volatiles, after harvest, storage

## Abstract

Potatoes are an important staple food with high yield potential and great nutritional value. Potassium (K) fertilisation can increase both tuber yield and quality, but its effects differ depending on the K fertilisation form. Potatoes are known to be chloride sensitive, since chloride ions can influence, for example, the starch content. Therefore, fertilisations shortly before planting using potassium sulphate (K_2_SO_4_) are often recommended instead of potassium chloride (KCl). However, the use of different fertilisation forms is contradictory, and the chloride sensitivity of potatoes remains unclear. To examine this issue in more detail, a 2-year field experiment using two cultivars, “Laura” and “Marabel,” was conducted. K fertilisation with 240 kg K_2_O as K_2_SO_4_ and KCl was applied, and the control remained unfertilised. Quality traits, including internal and external parameters, were analysed after harvest and after 5 months of storage at 6°C. The results revealed minor effects on yield, but the starch content and ascorbic acid concentration were reduced due to the KCl supply. Furthermore, the reducing sugar concentration in tubers increased during storage more after KCl compared to K_2_SO_4_ fertilisation. Moreover, volatile compounds were affected by the K fertilisation form, with higher levels of lipid-derived off-flavour compounds after KCl application. However, the effects of cultivation year, cultivar, and storage interacted with the influence of the fertilisation form. In summary, KCl fertilisation can disadvantageously influence several quality traits, but the use of potato cultivars should also be considered when recommending fertilisers.

## Introduction

Potatoes are an important staple food and, due to their high yield potential, contribute to global food security (Zaheer and Akhtar, 2016). Furthermore, the tubers have a great nutritional value, as they are rich in vitamin C, essential nutrients, such as potassium (K) and magnesium (Mg), and proteins of high biological value (Andre et al., 2014). Both, the yield and quality of potato tubers are determined by several factors, such as cultivar, environmental conditions, soil fertility, and fertilisation method (Rytel et al., 2013). Potatoes have a high demand for nutrients, which can be achieved by additional fertiliser applications to ensure yield and quality (Zaheer and Akhtar, 2016). K is one of the most important macronutrients, as it is essential for various plant physiological and metabolic processes, including starch synthesis and assimilate transport from source to sink (Zörb et al., 2014). Overall, K fertilisation has a great effect on tuber quality (Torabian et al., 2021). Thus, dry matter (DM) and starch contents are increased with K application, and the reducing sugar content is reduced. Moreover, the ascorbic acid concentration is enhanced, decreasing enzymatic discolouration and therefore improving processing characteristics (Mondy and Munshi, 1993). In addition, tubers that are sufficiently supplied with K have an increased cell turgor, are better protected against mechanical damages, and have enhanced storage properties (Naumann et al., 2020). Nevertheless, contrasting results are often found in relation to K fertilisation and tuber quality. For example, Westermann et al. (1994) found a reducing effect of K rates ranging between 112 and 448 kg potassium oxide (K_2_O) as potassium sulphate (K_2_SO_4_) or potassium chloride (KCl) on DM, while Khan et al. (2012) observed positive effects when applying 150 or 225 kg K_2_O as K_2_SO_4_ or KCl compared to an unfertilised control. In addition to the amount of K, however, the fertilisation form of K_2_O—as K_2_SO_4_ or KCl—is also important and can influence both yield and quality. Furthermore, the aspect of the cost of the fertiliser form is not completely irrelevant, since the production of KCl is considerably cheaper than potassium fertilisers containing sulphate, thus costs can be reduced during cultivation (Mikkelsen and Roberts, 2021).

Potatoes are assumed to be chloride sensitive (Mengel et al., 2001), since chloride fertilisers are reported to lead to reductions in tuber yield, DM, and specific gravity (e.g., Van Loon and Van Den Berg, 2003; Mohr and Tomasiewicz, 2012). Comparing fertilisation forms K_2_SO_4_ and KCl, sulphate was found to be superior to the chloride form for potato production in some studies. In a study by Sharma and Sud (2001) conducted on acidic and alluvial soils, rates of 75 and 150 kg K_2_O as K_2_SO_4_ increased DM, starch, and ascorbic acid content compared to the same rates of K_2_O as KCl. However, the positive effects of the sulphate component on, e.g., DM depend on the studied cultivars. Increased DM and starch content due to fertilisation with K_2_SO_4_ were also observed by Kumar et al. (2004) applying 124.5 kg K_2_O as K_2_SO_4_ and Manolov et al. (2016) fertilising with 100 and 200 kg K_2_O as K_2_SO_4_, respectively. Adverse effects on the observed parameters are mostly attributed to chloride ions in the soil solution (Torabian et al., 2021). KCl fertilisation is presumed to cause a higher osmotic potential compared to K_2_SO_4_, which leads to higher water uptake and growth rates, resulting in competition between shoots and tubers for assimilates (Koch et al., 2020). However, similar to K rates, contrasting results on quality are found in relation to the K fertiliser form. Davenport and Bentley (2001) and Khan et al. (2012) found that starch content and specific gravity were unaffected after KCl fertilisation. A recent study by Hütsch et al. (2018) investigated the different effects of K_2_SO_4_ and KCl treatments in pot experiments (soil and hydroponic), demonstrating that neither yield nor tuber quality was adversely affected by KCl compared to K_2_SO_4_ under the experimental conditions. The explanation for the differing results could be the cultivation method, cultivar-specific responses to chloride, agricultural management practices, including irrigation, and the general water supply by precipitation, since chloride is prone to leaching (Geilfus, 2019).

In the food industry, KCl is used as a salt substitute, e.g., in soups, sauces and salty snacks, but is reported to cause an unwanted metallic bitter taste (Greer et al., 2020). Thus, whether a KCl supply could also have a disadvantageous influence on the formation of volatile compounds determining potato aroma is unclear. To date, there have been few studies on the influence of fertilisation on potato aroma. It has been reported that individual volatiles are positively influenced by K fertilisation (Khan et al., 1977; Fischer, 1991). However, it has not yet been investigated whether the fertilisation form of KCl and K_2_SO_4_ have an influence on the volatile compounds. Considering the contradictory results on quality traits and different research approaches, potato chloride sensitivity remains unresolved. Furthermore, the examined literature focuses on often discussed quality traits, such as DM, starch, and ascorbic acid content. The objectives of this study were to investigate a more comprehensive set of parameters, including internal quality traits, such as starch, DM, reducing sugar, and sucrose content, as well as concentrations of amino acids, proteins, and ascorbic acid, and external quality traits, such as skin fracturability and thumbnail cracking, and how they are affected by K_2_SO_4_ compared to KCl fertilisation. Furthermore, not only freshly harvested tubers were investigated, but quality parameters were also analysed after 5 months of storage to elucidate the possible effects of fertilisation form during storage. In addition to the common parameters studied as potato quality traits, volatile analyses were performed to detect the possible effects of fertilisation form on volatile compounds. Moreover, different tuber parts, including the stem end, middle part, bud end, skin, and flesh, were analysed separately, presuming that fertilisation form might affect quality parameters within the tuber differently. Therefore, a 2-year field experiment in 2019 and 2020 was conducted using two table potato cultivars, “Marabel” and “Laura” to elucidate the effects of fertilisation forms K_2_SO_4_ and KCl on potato quality. We hypothesised that (I) KCl supply has adverse effects on tuber quality traits compared to K_2_SO_4_ fertilisation, (II) tuber parts are affected differently by fertilisation form, (III) KCl supply has adverse effects on volatile compounds, and (IV) fertilisation form additionally influences quality traits during 5 months of storage.

## Materials and Methods

### Field Experiments 2019 and 2020

In 2019, a field experiment with potato cultivar Marabel was conducted at the experimental station of the University of Goettingen, involving two different K treatments and one unfertilised control (Supplementary Figure S1). Marabel is a medium-early table potato with a predominantly waxy cooking type. The experiment included a block design with four plots, each for one of the K treatments (8 x 3 m) and four plots for the control (4 x 3 m). In each plot, four rows of potatoes were planted with a spacing of 30 cm. For analysis, only the inner two rows were sampled to exclude side effects between the fertilisation forms. Soil samples were taken before fertilisation to evaluate the soil mineral status (Supplementary Table S1). K fertilisers were applied before planting, containing 240 kg K_2_O as K_2_SO_4_ (30% K_2_O, 10% MgO, and 42.5% SO_3_) and KCl (40% K_2_O, 6% MgO, 4% Na_2_O, and 12.5% SO_3_). In the text, tubers fertilised with KCl or K_2_SO_4_ are referred to as KCl or K_2_SO_4_ tubers. Furthermore, nitrogen was applied as calcium ammonium nitrate (8% Ca and 27% N), phosphorous as triple superphosphate (46% P_2_O_5_), and magnesium as magnesium oxide *via* kieserite (25% MgO, 50% SO_3_; Supplementary Table S2). The control remained unfertilised. The growing season ranged from 25th April to 27th August. The average day temperature and precipitation rate of the trial site are shown in Supplementary Figure S2.

In 2020, a field experiment was set up with the same two K fertilisation treatments in each of four blocks (Supplementary Figure S3). Additionally, two more blocks for the control treatment were planted, and a second cultivar, Laura, was established within the same experimental design. Similar to Marabel, Laura is a medium-early table potato with a predominantly waxy cooking type; however, Laura has strong yellow flesh and red skin. The growing season ranged from 29th April to 7th September, and the average day temperature and precipitation of the trial site can be obtained from Supplementary Figure S4.

### Sample Preparation and Storage Conditions

**Figure 1:**
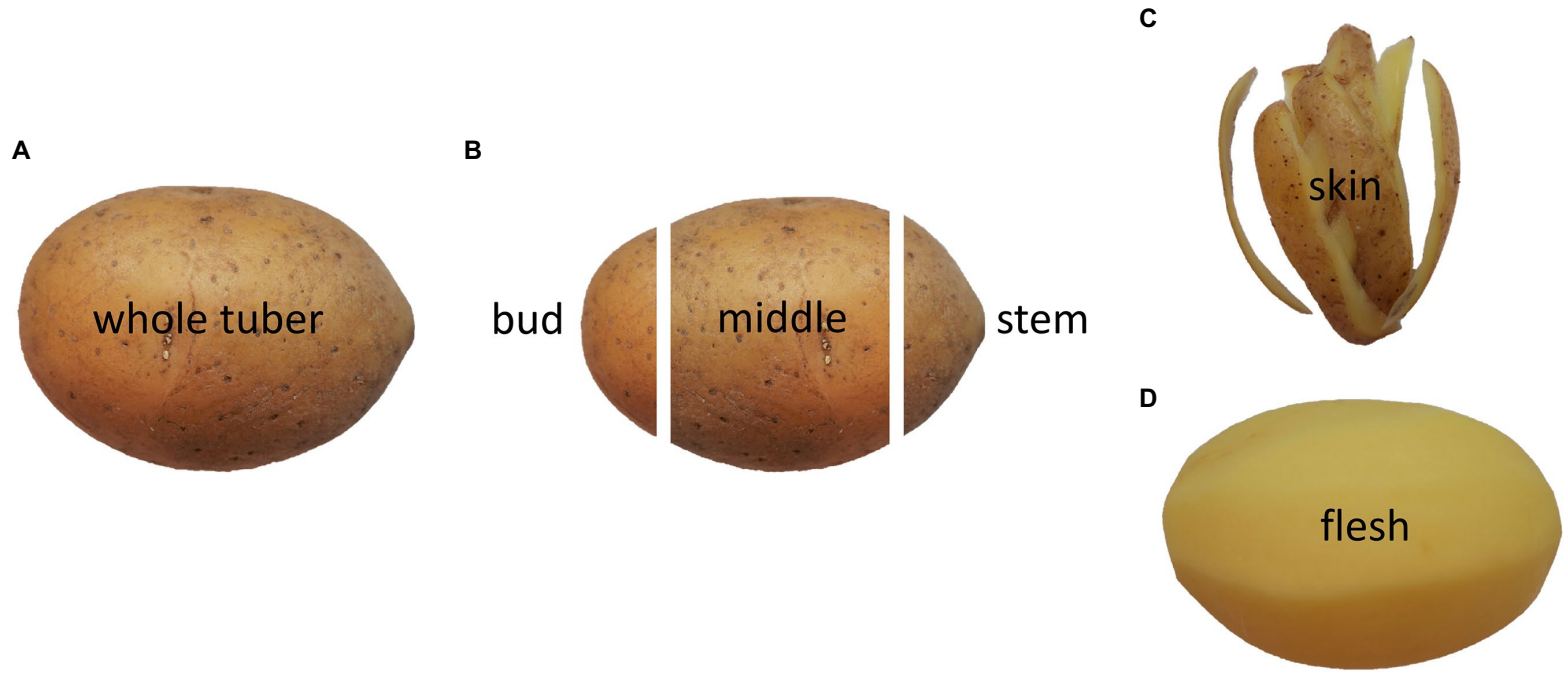
Illustration of tuber parts used for analyses showing whole tuber **(A)**, bud end, middle part, stem end **(B)**, skin **(C)**, and flesh **(D)**.

After harvesting, the tubers were first stored in boxes at 6°C with 95% humidity. For preparation, tubers were divided into stem end, middle part, bud end, skin, and flesh, as illustrated in Figure 1. The skin is a 2 mm thick layer, including the periderm and part of the cortex, and the flesh is defined as the pith and perimedullary zone. The stem end was characterised as a 15% thick part of the tuber where the stolon was adjusted, including part of the skin. The bud end also made up a 15% thick part of the tuber where the eyes are clustered, including part of the skin. The middle part covers 70% of the tuber, is located between the stem and bud ends, and contains the perimedullary zone and the pith. Additionally, all analyses were performed on the whole tuber, which included all the above-mentioned parts. Five tubers for stem end, middle part, and bud end, five tubers for skin and flesh, and five whole tubers were cut into pieces and freeze-dried (EPSILON 2-40, Christ, Osterode am Harz, Germany). Thumbnail cracking and skin fracturability were evaluated on the entire tuber. All other analyses were performed on the different tuber parts. Measurements were conducted after harvest within 6 weeks for 2019 and 2020 and cultivars Marabel and Laura. After a 5-month storage period under the above-mentioned conditions, all following described analyses were conducted again, except yield, starch yield, thumbnails, and skin fracturability.

### Yield, Starch Content, and Starch Yield

The yield was determined by weighing the tubers from the harvested rows and calculating the weight per hectare. The starch content was measured only for the flesh part of the tuber. Analyses were performed on 1 g freeze-dried material, using hydrochloric and tungstophosphoric acid, and optical rotation was examined with a polarimeter (Zeiss, Oberkochen, Germany) at 589 nm as described in Koch et al. (2019b). Using data on yield and starch content, the starch yield was calculated.

### DM, Mineral, and Sugar Concentrations

Around 10 g of fresh shredded material from each tuber part was weighed into petri dishes and dried at 65°C for 21 h and three additional hours at 105°C. Afterwards, samples were weighed again, and DM content was calculated. For the determination of mineral concentrations, 100 mg freeze-dried material was used, prepared, and measured according to Koch et al. (2019a).

Reducing sugars (glucose and fructose) and sucrose were determined using 750 mg freeze-dried tuber material. The procedure was done as described in Koch et al. (2019a) and sugars were measured by HPLC (LC-2000 Plus, Jasco, Pfungstadt, Germany).

### Chloride and Sulphate Ion Concentrations

To determine chloride and sulphate ions, 25–30 mg of freeze-dried tuber and leaf material were weighed into Eppendorf tubes, filled with 1 ml distilled water and shaken for 3 h. After shaking, samples were centrifuged at 23°C and 25,000 × *g* for 30 min. Resulting supernatant was transferred to a sample concentrator (PES, MWCO 3 kD, VWR, Monroeville, PA, United States) and centrifuged at 23°C and 8,000 × *g* for 60 min again. Once more, samples were filtered and a 20-fold dilution with a sodium carbonate/sodium hydrogen carbonate (Na_2_CO_3_/NaHCO_3_) eluent was prepared. Determination was performed with a Metrosep A Supp 17 150/4.0 column (Metrohm, Filderstadt, Germany) at a flow rate of 0.7 ml/min using the Metrohm ECO IC system.

### Ascorbic Acid Concentration

The same number of tubers for DM, mineral, and sugar concentrations for each part was used for the measurement of ascorbic acid. Five grams of the fresh material were weighed into measuring cylinders, and 20 ml of 5% metaphosphoric acid [(HPO_3_)_3_] was added. The mixture was then homogenised with a vortex mixer (T18 digital Ultra Turrax, IKA, Staufen, Germany), filled to 50 ml with distilled water, and transferred through a filter (Filter paper MN 616 ¼, Macherey-Nagel GmbH & Co. KG, Düren, Germany) into conical flasks. Ten millilitres of the filtrate were titrated three times against the 2,6-dichlorophenolindophenol (DIP) solution (0.2 g of DIP in 1000 ml distilled water) until the solution changed from colourless to light pink. For Laura, only flesh could be measured for ascorbic acid content, since the colour change was not visible in parts, including the red skin.

### Evaluation of Thumbnail Cracks and Measurement of Skin Fracturability

To evaluate the resistance to form thumbnail cracks, tubers were damaged in a drum, as described by Koch et al. (2019b). Subsequently, the tubers were stored for 7 days at room temperature, and the evaluation of thumbnail cracks was performed using a grading system from 1 (severe thumbnail occurrence) to 9 (no thumbnails) according to Koch et al. (2019b).

To assess tuber skin fracturability, 10 tubers from each plot were analysed using a texture analyser (Stable Micro Systems Ltd., TA.XT.plus, Godalming, United Kingdom) and measuring the maximum force (in Newton) required for breaking the skin. Measurements were performed with a stamp of 5 mm Ø and a speed of 5 mm^−s^, penetrating the tuber skin and the subjacent flesh to a depth of 10 mm.

### Total Free Amino Acid and Protein Concentration

To measure total free amino acids and protein concentration, an ethanolic extraction was performed on 10 mg freeze-dried tuber material, as described in Chea et al. (2021). The measurement of free amino acids was also performed according to Chea et al. (2021). The remaining pellets after ethanolic extraction were further treated for protein determination. The pellets were resuspended with 400 μl of 0.1 M NaOH, heated for 30 min at 95°C, and shaken on a heating block. After cooling to room temperature, samples were centrifuged for 10 min at 10,600 × *g*. The protein concentration was determined according to the method by Bradford (1976) modified by Zor and Selinger (1996) using a Bradford protein kit (Merck, Darmstadt, Germany) and bovine serum albumin as standard.

### Volatile Compounds

Analyses of volatile compounds were performed on 2-cm-thick vertical slices from the middle of each of the five tubers. The slices were cut into small pieces and either further prepared raw or boiled in advance. Boiling was performed for *ca.* 10 min in a beaker filled with 200 ml water. For further preparation, 50 g of either raw or boiled tuber pieces were weighed into separate beakers. Following sample preparation, identification of the volatiles, and subsequent measurements on a gas chromatograph (GCMS-TQ8040, Shimadzu Deutschland GmbH, Duisburg, Germany), including device settings, were performed according to Kanski et al. (2020). The sample vials were stored until analysis at −20°C. The amount of individual aroma compounds is expressed as the relative abundance of GC peak area unit (A), as described in Duckham et al. (2001), where A = a_1_/a_2_ wherein a_1_ = peak area of sample component and a_2_ = peak area of internal standard (octanol). The relative abundance was further used to calculate fold changes, comparing (I) raw tubers after harvest with raw tubers after 5 months of storage and (II) raw tubers after harvest with cooked tubers after harvest.

### Statistical Analyses

Statistical analysis was performed using the Statistical Package for the Social Sciences (SPSS) software version 25 (IBM Statistics, Armond, NY, United States). Data were checked for normal distribution and homoscedasticity before performing ANOVA to detect differences between the mean values of the treatments, followed by Tukey’s *post hoc* test. All statistical tests were performed at a significance level of *p* < 0.05 if not described differently. The fertilisation effect describes all three treatments, including the unfertilised control after harvest. Differences between fertilisation form (K_2_SO_4_ and KCl) were analysed separately and are described in the respective text sections. The effect of year was considered only for the after harvest data of Marabel in 2019 and 2020. Cultivar effects refer to the differences between Marabel and Laura in 2020. Storage effects refer to the data from both cultivars and both years. Interactions between the factors are shown in the supplement (Supplementary Tables S3, S4) and were only indicated when one of the parameters measured revealed interaction effects.

## Results

### Tuber and Starch Yield

Despite the assumption that the fertilisation form would influence individual tuber parts differently compared to whole tubers, no differences were found. Therefore, the following section describes the results of the entire tubers only. The results for the individual tuber parts of each parameter can be obtained from the supplementary material (Supplementary Tables S5–S8). Tuber yield was significantly influenced by fertilisation treatments and cultivation year, but not by cultivar (Table 1). Moreover, fertilisation treatment and cultivar influenced the starch content, but year and storage showed no effects. Based on yield and starch content, the calculated starch yield was not affected by fertilisation or cultivar but showed differences between cultivation years for Marabel. K_2_SO_4_ fertilisation increased Marabel yield by 4%–18% and KCl supply by 25%–28% compared with the control, depending on year; however, KCl treatment reduced starch content by 9%–14%, while K_2_SO_4_ showed reductions of only 4%–10%. The starch content was reduced more after storage in KCl compared to K_2_SO_4_ tubers. However, the differences between K_2_SO_4_ and KCl supply in yield and starch content were greater in 2019 and minor in 2020 (Table 1).

**Table 1:** Yield (t ha^−1^), starch content (% FM), and starch yield (t ha^−1^) depending on fertilisation form, cultivar, year, and storage.

Year	Cultivar	Parameter	Harvest			Storage (5 months)		
			Control	K_2_SO_4_	KCl	Control	K_2_SO_4_	KCl
2019	Marabel	Yield	35.6 ± 0.1^b^	42.3 ± 2.2^a^	44.5 ± 2.8^a^	n.d.	n.d.	n.d.
		Starch content	17.1 ± 0.1^a^	15.4 ± 0.5^b^	14.7 ± 0.7^b^	17.1 ± 1.1^a,#^	14.8 ± 0.9^b^	13.2 ± 0.2^c^
		Starch yield	6.1 ± 0.1^a^	6.5 ± 0.5^a^	6.5 ± 0.5^a^	n.d.	n.d.	n.d.
2020	Marabel	Yield	52.7 ± 11.0^a^	55.1 ± 6.5^a^	67.8 ± 5.0^a^	n.d.	n.d.	n.d.
		Starch content	16.2 ± 0.5^a^	15.4 ± 0.7^a^	14.7 ± 1.2^a^	15.7 ± 0.2^a^	14.0 ± 1.1^a,b^	13.2 ± 1.3^b^
		Starch yield	8.5 ± 1.7^a^	8.5 ± 1.0^a^	10.0 ± 1.0^a^	n.d.	n.d.	n.d.
	Laura	Yield	53.6 ± 7.9^a^	59.2 ± 6.5^a^	58.1 ± 4.7^a^	n.d.	n.d.	n.d.
		Starch content	18.4 ± 0.6^a^	16.8 ± 0.6^ab^	16.0 ± 1.2^b^	18.3 ± 0.7^a^	15.8 ± 1.1^b^	15.6 ± 0.6^b^
		Starch yield	9.9 ± 1.6^a^	10.0 ± 1.3^a^	9.2 ± 0.6^a^	n.d.	n.d.	n.d.

Significances—Yield: fertilisation* year*** cultivar n.s. Starch content: fertilisation*** year n.s., cultivar*** storage n.s. Starch yield: fertilisation n.s., year*** cultivar n.s. Mean ± SD (*n* = 4), *** *and* *** for *p* < 0.05, 0.01, and 0.001, respectively. Small letters = differences between the fertilisation treatments within one parameter after harvest or 5 months of storage. Starch content was measured on tuber flesh only. Effect of year considers differences of tubers after harvest between 2019 and 2020 for Marabel, cultivar effect describes differences between Laura and Marabel in 2020, and storage effects refer to data of both cultivars and both years. n.d., Not determined. n.s., Not significant.

### Tuber Quality

**Figure 2:**
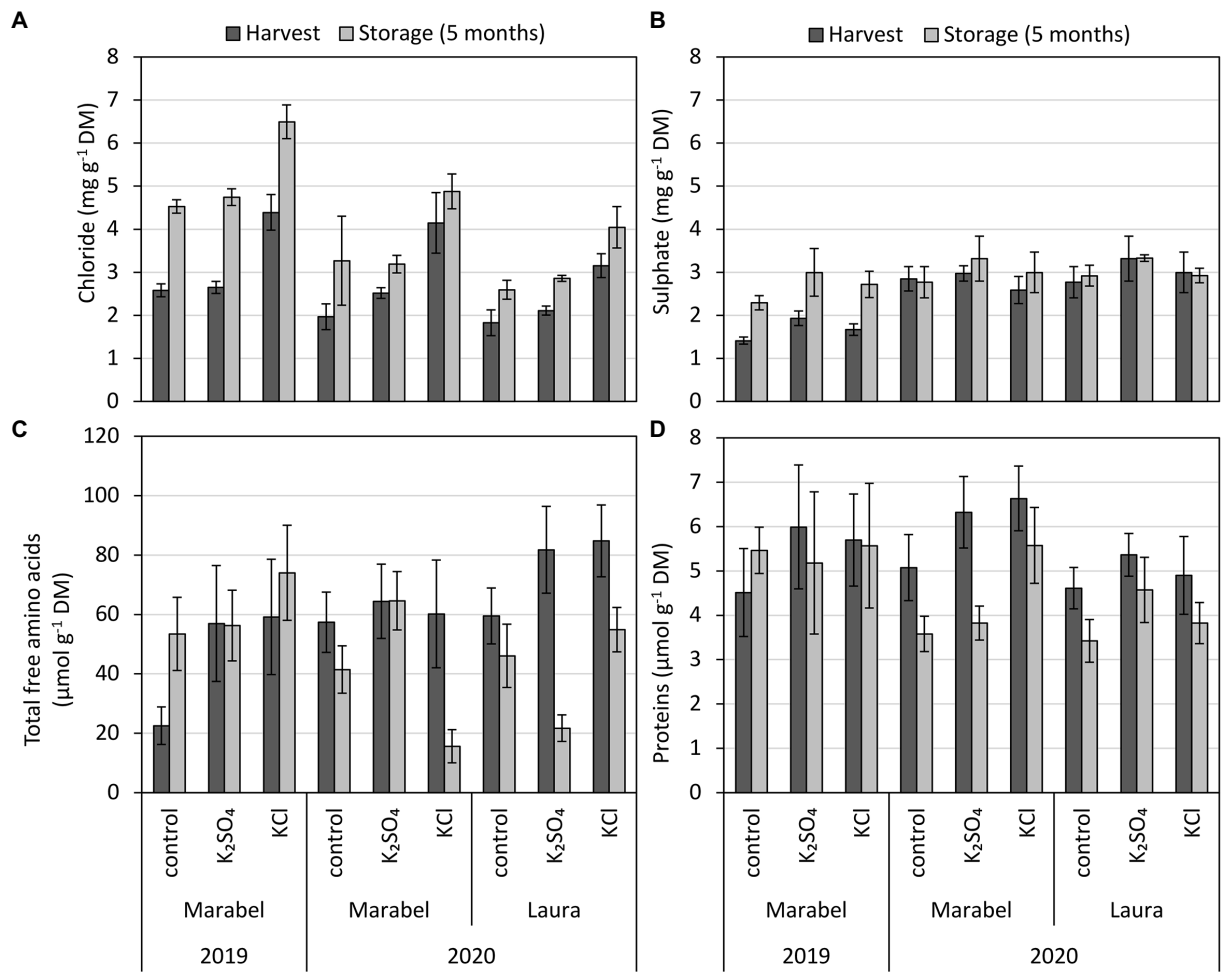
**(A)** Chloride, **(B)** sulphate, **(C)** total free amino acids, and **(D)** protein concentration of entire tubers depending on year, cultivar, fertilisation form, and storage. Mean ± SD (*n* = 4). *, **, and *** for *p* < 0.05, 0.01, and 0.001, respectively. n.s., Not significant. Effect of year considers differences of tubers after harvest between 2019 and 2020 for Marabel, cultivar effects describe differences between Laura and Marabel in 2020, and storage effects refer to data of both cultivars and both years. Chloride: fertilisation***, year n.s., cultivar n.s., storage***; sulphate: fertilisation n.s., year***, cultivar n.s., storage***; amino acids: fertilisation**, year n.s., cultivar***, storage**; and proteins: fertilisation***, year n.s., cultivar*, storage**.

The K concentration was influenced by the fertilisation treatment, revealing more K in the KCl and K_2_SO_4_ tubers compared to the control (Figure 2). Within the tuber parts, the K concentration increased from stem to bud end in both cultivars and years (Supplementary Table S7). The significant interaction effects of cultivar, fertilisation, storage, and year for the quality parameters in tubers are presented in the supplement (Supplementary Tables S3, S4).

The chloride concentration was strongly affected by fertilisation and storage, but cultivar and year effects were not observed. Depending on the fertilisation form, the highest chloride concentrations were measured in the KCl tubers (Figure 2A) and KCl tuber parts (Supplementary Tables S6, S7) compared to K_2_SO_4_ tubers. After 5 months of storage, the chloride concentration increased in both years and cultivars (Figure 2A). In contrast, the sulphate concentration did not show any effect of fertilisation but was influenced by the year, with higher concentrations in 2020 than in 2019. Cultivar effects were not detected, but similar to chloride, concentrations increased during storage, but to a lesser extent (Figure 2B).

Total free amino acid and protein concentrations increased by fertilisation treatments compared to the control. Differences in total free amino acids and proteins between the KCl and K_2_SO_4_ tubers, however, were not observed. Cultivar effects were greater in amino acid concentrations compared to proteins, revealing higher concentrations after harvest in Laura than in Marabel. The opposite was found with the protein concentration. Overall, a year effect was not observed for both parameters, but storage decreased amino acid, as well as protein, concentration in all three treatments and both cultivars in 2020, except for Marabel in 2019 (Figures 2C,D).

**Figure 3:**
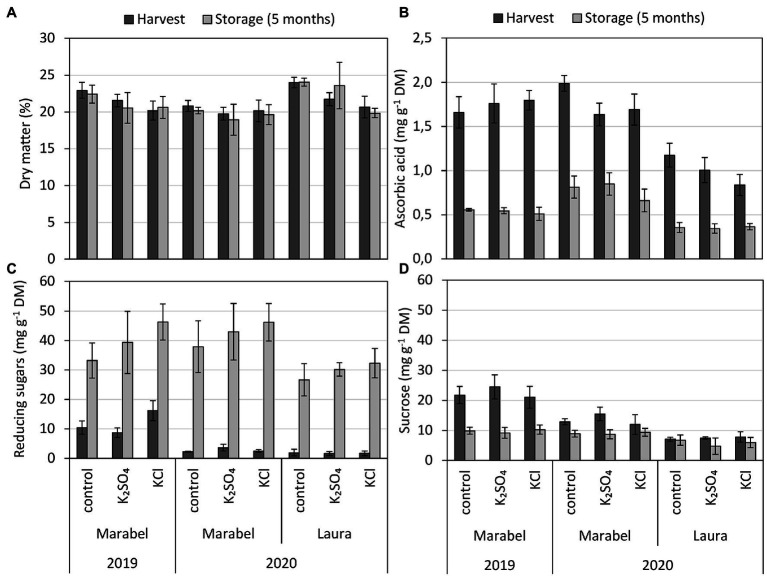
**(A)** Dry matter, **(B)** ascorbic acid (flesh), **(C)** reducing sugar, and **(D)** sucrose concentration of entire tubers depending on year, cultivar, fertilisation form, and storage. Mean ± SD (*n* = 4). *, **, and *** for *p* < 0.05, 0.01, and 0.001, respectively. n.s., Not significant. Effect of year considers differences of tubers after harvest between 2019 and 2020 for Marabel, cultivar effects describe differences between Laura and Marabel in 2020, and storage effects refer to data of both cultivars and both years. DM: fertilisation**, year**, cultivar***, storage n.s.; ascorbic acid: fertilisation**, year*; cultivar***; storage***; Red. sugars: fertilisation***, year***, cultivar***, storage***; and sucrose: fertilisation n.s., year***, cultivar***, storage***.

The year, cultivar, and fertilisation treatments significantly affected the DM content (Figure 3A). With fertilisation, DM decreased by 5%–10% in K_2_SO_4_ tubers and by 13%–16% in KCl tubers compared to the control. Compared to Marabel, Laura’s DM was significantly higher in 2020, with the lowest content in KCl tubers. However, the K fertilisation form showed no significant difference. After storage, DM was slightly decreased, but revealed no significant differences compared to values after harvest. Skin fracturability and thumbnails were not affected by fertilisation but showed cultivar effects within skin fracturablity and an effect of year within thumbnail score (Supplementary Figure S5).

The ascorbic acid concentration was strongly influenced by cultivar, storage, and fertilisation and minorly affected by year. The highest concentrations were measured for both cultivars and both years in the control, and the lowest concentrations contained KCl tubers (Figure 3B). Comparing the cultivars, the concentration of ascorbic acid was one-third lower in tubers of Laura compared to tubers of Marabel. Furthermore, Laura’s KCl tubers revealed 5 mg/100 g FW less ascorbic acid compared to K_2_SO_4_, which represents 5 mg per 100 g FW (Supplementary Figure S6), highlighting significant differences between K fertilisation forms. After storage, ascorbic acid concentration decreased by 50%–72%, showing the strongest decrease in KCl tubers of Marabel in 2019 and 2020 (Figure 3B).

Similar to ascorbic acid, the concentration of reducing sugars was affected by cultivar, storage, fertilisation, and additionally by year. With the KCl supply, the concentration of reducing sugars increased in Marabel in 2019, but not in 2020, where the concentration in K_2_SO_4_ tubers was higher (Figure 3C). Overall, no significant differences between K fertilisation forms were observed. Compared to 2019, the reducing sugar concentration was reduced by about 80%. During 5 months of storage, their concentration increased strongly by a factor of three in 2019 and even by a factor of 10 in 2020 for both cultivars. Concentration, however, was 30% lower after storage in Laura than in Marabel (Figure 3C).

The year, cultivar, and storage period significantly affected sucrose content in tubers. Fertilisation treatment, however, showed no significant effect but revealed higher contents of sucrose in K_2_SO_4_ tubers compared to the control, which was not noted for KCl tubers. In 2019, after harvest, the sucrose content in Marabel was 2-fold higher than in 2020 (Figure 3D). During storage in 2019, sucrose content decreased by one-half, while in 2020, the decrease was only about one-third. Overall, the sucrose content in Marabel was higher than in Laura (Figure 3D).

### Volatile Compounds in Boiled and Stored Tubers Affected by the K Fertilisation Form

A total of 19 volatile compounds were analysed and identified by name. Values represent fold changes of volatiles comparing raw and boiled tubers at harvest in 2020 (Table 2) and raw tubers at harvest to raw tubers after 5 months of storage (Table 3). Changes in raw and boiled fertilised tubers at harvest and after 5 months of storage compared to unfertilised control tubers are shown in the supplement (Supplementary Tables S9, S10).

**Table 2:** Fold changes of volatile compounds comparing raw and boiled potato tubers at harvest in 2020 depending on fertilisation and cultivar.

Volatile compound	Laura			Marabel		
	Control	K_2_SO_4_	KCl	Control	K_2_SO_4_	KCl
Pentanal	1.76	10.20	18.67	10.20	44.53	13.47
Hexanal	1.38	5.85	17.23	113.67	109.76	59.36
Heptanal	1.00	4.63	4.00	29.00	27.20	20.38
2-Methylbutanol	1.80	1.78	1.89	3.84	3.29	3.21
3-Methylbutanol	0.29	0.56	0.67	0.94	0.81	1.05
2-Pentylfuran	0.04	0.10	0.30	1.69	1.86	1.41
1-Pentanol	0.11	0.12	0.24	1.66	1.24	1.68
Octenal	0.64	0.39	0.62	3.27	2.77	4.04
2-Heptenal	1.09	2.12	3.07	38.11	29.02	28.21
Nonanal	0.21	2.00	1.33	3.29	9.82	3.33
2-Octenal	0.25	0.20	0.39	2.22	2.48	2.26
2-Isopropyl-3-methoxypyrazine	0.18	0.06	0.22	0.24	1.40	0.15
1-Octen-3-ol	0.08	0.06	0.13	0.87	1.05	0.85
Methional	0.00	0.00	0.00	0.01	0.14	0.05
2,4-Heptadienal	1.67	1.52	0.43	2.24	3.63	2.09
Decanal	1.16	1.17	2.92	15.21	17.40	13.90
2-Nonenal	0.71	0.77	0.61	0.71	0.68	0.61
2,4-Nonadienal	0.87	1.78	5.89	104.46	100.00	52.13
2,4-Decadienal	0.14	0.20	0.89	37.67	32.34	24.10

Values are expressed as relative fold changes comparing mean values of raw tubers at harvest with mean values of boiled tubers at harvest. Colour scale showing increase (>1 = red) or decrease (<1 = blue) of individual volatile compounds during boiling. *n* = 4.

**Table 3:** Fold changes of volatile compounds comparing raw potato tubers at harvest in 2020 with raw tubers after 5 months of storage at 6°C depending on fertilisation and cultivar.

Volatile compound	Laura			Marabel		
	Control	K_2_SO_4_	KCl	Control	K_2_SO_4_	KCl
Pentanal	0.36	0.80	4.89	0.00	1.20	0.00
Hexanal	0.91	0.75	6.87	0.65	0.91	0.16
Heptanal	24.19	89.50	74.67	112.60	130.00	112.43
2-Methylbutanol	0.75	1.13	1.45	1.35	1.38	1.52
3-Methylbutanol	2.36	3.56	1.67	1.11	1.14	1.11
2-Pentylfuran	1.48	1.09	1.93	1.22	1.10	0.58
1-Pentanol	1.29	1.12	1.95	1.38	1.20	0.77
Octenal	1.59	1.55	2.67	0.95	0.98	1.12
2-Heptenal	0.75	1.08	1.60	1.16	1.19	0.77
Nonanal	0.18	1.50	1.00	1.00	0.73	1.33
2-Octenal	1.96	1.22	2.31	1.07	1.03	0.65
2-Isopropyl-3-methoxypyrazine	0.79	0.53	0.89	0.80	0.73	0.51
1-Octen-3-ol	1.53	1.32	1.55	1.34	1.32	1.03
Methional	0.94	0.26	0.24	0.10	0.09	0.08
2,4-Heptadienal	9.69	6.52	4.82	4.14	5.83	3.36
Decanal	0.83	0.63	2.03	1.33	1.55	0.39
2-Nonenal	1.73	0.89	0.47	0.45	0.40	0.41
2,4-Nonadienal	1.01	0.66	4.28	2.31	1.56	0.13
2,4-Decadienal	1.81	0.78	7.75	5.61	2.79	0.30

Values are expressed as relative fold changes comparing mean values of tubers at harvest with mean values of tubers after 5 months of storage. Colour scale showing increase (>1 = red) or decrease (<1 = blue) of individual volatile compounds during storage in raw tubers. *n* = 4.

During boiling, changes in individual volatile compounds were observed. Pentanal and hexanal strongly increased in fertilised tubers of Laura, with a greater increase in KCl compared to K_2_SO_4_ tubers (Table 2). In Marabel, both volatiles increased in all treatments, with a 44.53-fold increase of pentanal in K_2_SO_4_ tubers. Hexanal increased more than 100-fold in the control and K_2_SO_4_ tubers and only 59.36-fold after KCl fertilisation. Furthermore, heptanal increased 4-fold in fertilised tubers of Laura, whereas it increased in Marabel up to 29-, 27.20-, and 20.38-fold in control, K_2_SO_4_ and KCl tubers, respectively. Increases were also observed in 2-methylbutanol, with a 3-fold increase in all three treatments in Marabel. Volatile compounds, namely 2-pentylfuran, 1-pentanol, 2-isopropyl-3-methoxypyrazin, 1-octen-3-ol, and methional, decreased during boiling, especially in Laura. Decanal, 2,4-nonadienal, and 2,4-decadienal increased during boiling, mostly in Marabel, for all treatments. In Laura, decanal and 2,4-nonadienal increased in the KCl tubers only.

The volatile compounds of raw potatoes were influenced by 5 months of storage. Pentanal and hexanal increased strongly in Laura during storage in KCl compared to K_2_SO_4_ tubers and the control, where both compounds decreased. A reduction in both volatiles was detected in Marabel. The highest influence of storage was observed in heptanal in both cultivars and all treatments, with up to a 130-fold increase in Marabel K_2_SO_4_ tubers. Furthermore, 2-isopropyl-3-methoxypyrazin and methional decreased during storage in all treatments and for both cultivars, with greater reductions in methional due to fertilisation. In Laura, 2,4-heptadienal increased 9.69-, 6.52-, and 4.82-fold in the control, K_2_SO_4_, and KCl tubers, respectively, while in Marabel, increases of 4.14-, 5.83-, and 3.36-fold, respectively, were observed (Table 3). In contrast, 2,4-nonadienal and 2,4-decadienal increased 4.28- and 7.75-fold, respectively, in KCl tubers of Laura, while both volatiles decreased to 0.13 and 0.30 in KCl tubers of Marabel.

## Discussion

### Effect of Fertilisation Form on Tuber Yield and Quality

In potato production, K is known to influence many quality parameters. Contradictory results in tuber quality regarding K fertilisation itself and the K fertilisation form have been found in the last decades, and the chloride sensitivity of potatoes is still unresolved (Khan et al., 2012; Hütsch et al., 2018).

Therefore, a 2-year field experiment was conducted to elucidate the response of a wide range of quality parameters of two potato cultivars to K_2_SO_4_ and KCl fertilisation. The results of the tuber parts did not differ from those of the whole tuber in relation to the fertilisation form. Therefore, in this section, the results of the whole tubers were discussed, and hypothesis II, tuber parts are affected differently by fertilisation form, was rejected.

The fertilisation increased tuber yield compared to the control. This has already been found in some studies (Li et al., 2015; Soratto et al., 2020; Ali et al., 2021). The K fertilisation form, however, did not reveal significant differences but showed some higher tuber yields in Marabel in both years after KCl fertilisation. Due to the chloride component, the osmotic potential is reduced, leading to increased water uptake, which is responsible for higher growth and yield rates (Beringer et al., 1990; Koch et al., 2020). However, improved water uptake could be the reason for lower starch concentrations in KCl tubers due to a dilution effect (Mohr and Tomasiewicz, 2012), which was also noted in our study, since the water content in KCl was slightly, but not significantly, higher compared to K_2_SO_4_ tubers. However, the calculated starch yield was not affected by the fertilisation form because it was compensated by the higher yield potential in KCl tubers. Starch content decreased with fertilisation in both years and for both cultivars. These observations were not in line with Khan et al. (2012), who found no changes due to increasing K rates when applying KCl, but compared to the control, K_2_SO_4_ supply increased starch content. However, with increasing K_2_SO_4_ rates, the starch content remained unaffected. Nevertheless, the highest rate of K applied in their study was 225 kg K_2_O, which was lower than the fertilised rates used in the present study. Similarly, starch content usually increases with K application when K is deficient in soils because K is involved in the activation of the enzyme starch synthase, which is responsible for starch synthesis by converting ADP–glucose into long-chain starch molecules (Mengel et al., 2001). Higher doses of K can, in contrast, decrease the starch content, which can be explained by the increased water content maintaining cell turgor pressure (Westermann et al., 1994) additionally leading to a dilution effect. This might have been the case in the present study, since the soil was not deficient in K (Supplementary Table S1).

The results of the ion measurements showed that more chloride accumulated in the KCl tubers and more sulphate in K_2_SO_4_ tubers. Chloride ions showed a stronger increase in KCl tubers than sulphate in K_2_SO_4_ tubers. Chloride ions are characterised by their higher mobility within plants compared to sulphate ions, which might explain the higher concentrations in the respective treatments. Similar results in tobacco plants (*Nicotiana tabacum* L. var. Habana) were found by Franco-Navarro et al. (2016), concluding Cl^−^ are more included in biological processes, such as electrical charge balance of K^+^, than SO_4_^−^. Due to elevated chloride uptake, sulphate might be inhibited in its uptake (Zehler et al., 1981), explaining the slightly lower concentrations in the skin and flesh. However, concentrations of both ions were attributed to year effects, e.g., higher water availability or soil conditions. Cl^−^ is especially prone to leaching, explaining the lower concentrations in Marabel tubers in 2020 compared to 2019, which is related to higher precipitation rates in 2020 (Supplementary Figures S2, S4).

As an important quality aspect of processed potatoes, such as French fries and chips, the DM content should be high, since it positively affects the texture of potato products. In this study, the DM content decreased with fertilisation compared to the control and was higher in Laura than in Marabel. This is in line with result from Simson et al. (2016), who found decreasing DM contents in fertilised tubers and differences between cultivars. In the present study, DM content of KCl tubers of Marabel in 2019 and Laura in 2020 was the lowest. Kumar et al. (2007) also observed reduced DM in KCl compared to K_2_SO_4_ tubers. However, comparing K fertilisation forms, contradictory results on DM were observed in previous studies, revealing adverse influences of KCl rates ranging within 112–448 kg K_2_O (Westermann et al., 1994) or positive effects of 150 and 225 kg K_2_O as KCl (Khan et al., 2012). Beside fertiliser form (KCl or K_2_SO_4_), differences between these studies are referred to K soil status, and cultivar. The higher DM content in Laura in our study was connected to a higher starch content.

Furthermore, in comparison with K_2_SO_4_ fertilisation, KCl had an adverse effect on the concentration of ascorbic acid in both years and for both cultivars, which could indicate a reducing effect of chloride ions on ascorbic acid synthesis. In tubers, ascorbic acid has an important influence on the delay of enzymatic discoloration during processing (Mondy and Munshi, 1993), which might be impaired due to Cl^−^ when using KCl fertilisation. Presumably, Cl^−^ could affect precursors of ascorbic acid in the biosynthetic pathway. Manolov et al. (2016) confirmed a reducing effect of 600 kg K_2_O as KCl, which was stronger compared to the same rate of K_2_SO_4_, but represents excessive amounts of K. Mondy and Munshi (1993) could also show reducing effects of KCl on the ascorbic acid concentration for cultivar “Pontiac,” but in the same study, cultivar “Ontario” showed contrasting results, highlighting cultivar-specific changes in ascorbic acid content. Differences in ascorbic acid concentrations due to K fertilisation form therefore depend strongly on the nutritional status of the soil and the soil type, environmental conditions, and cultivars (Hamouz et al., 2009; Samaniego et al., 2020). Cultivar effects were also observed in the present study, with a higher concentration of ascorbic acid in Marabel than in Laura. These results are in line with several publications (e.g., Valcarcel et al., 2016; Hamouz et al., 2018). However, the effects of year in this study, including location and environmental conditions, were of minor importance and were dominated by cultivar effects. This is in contrast to Hamouz et al. (2007), demonstrating contributions of high temperatures and low precipitation on increasing the ascorbic acid contents.

Another important parameter that significantly influences the quality of potatoes is reducing sugars. Their concentrations affect the processing quality and nutritional value of tubers (Kumar et al., 2004). Participating in the Maillard reaction with amino acids, sugars contribute greatly to the colour and taste development of potato products (Wiberley-Bradford et al., 2014). However, they are precursors in acrylamide formation during frying processes, which are considered carcinogenic and neurotoxic for humans (Zörb et al., 2014). Similar to ascorbic acid, the concentration of reducing sugars was affected by fertilisation, cultivar, and year in the present study. The concentration of reducing sugars was highest in KCl tubers of Marabel in 2019 compared to the control and K_2_SO_4_, but in 2020, it was higher in K_2_SO_4_ tubers compared to other treatments. Overall, no significant differences were observed between the effects of K_2_SO_4_ and KCl supplies. Previous studies have shown that K application leads to a decrease in reducing sugar synthesis and, therefore, concentration (Gerendás et al., 2007; Bansal and Trehan, 2011). Due to sufficient K in plant tissues, only lower concentrations of reducing sugars are needed to maintain osmotic homeostasis (Gerendás et al., 2007; Marschner and Rengel, 2012). However, in the present study, this effect was only partly shown in K_2_SO_4_ tubers of Marabel in 2019 and in K_2_SO_4_ and KCl tubers of Laura. The differences in Marabel between 2019 and 2020 showed that the concentration of reducing sugars was influenced by the year and, therefore, differing environmental conditions. These can include nutrient supply with nitrogen (N) or sulphur (S) in the soil or environmental conditions, such as heat or moisture stress, during the growing period, which can increase the accumulation of reducing sugars (Kumar et al., 2004). Thus, higher average temperatures and lower precipitation rates during the growing season in 2019 compared to 2020 could explain the increased levels of reducing sugars. Overall, considering the usage of the studied potato cultivars, the quality determinant of reducing sugar after harvest is of less importance, since concentrations are low, but should be considered when producing tubers for processing.

After harvest, the most abundant sugar found in tubers was sucrose, which was also reported by Duarte-Delgado et al. (2016). There was no effect observed related to fertilisation, but in 2019, higher concentrations of sucrose were measured compared to 2020 for Marabel, revealing environmental influences on sucrose content. Moreover, the sucrose concentration indicates tuber maturity, which is related to lower concentrations (Kumar et al., 2004). This might explain the differing results based on cultivation year, since tubers in 2019 were harvested 2 weeks earlier due to weather conditions and, therefore, were likely less mature. These results are supported by Heltoft et al. (2017), who showed a decreasing sucrose content during the last 3 weeks before harvest. Furthermore, the sucrose concentration was lower in Laura than in Marabel in 2020, which shows cultivar-specific responses of sucrose accumulation and might be related to different maturity periods of the cultivars (Kumar et al., 2004).

The fertilisation increased the concentration of total free amino acids, which is likely attributed to the N supply. Amino acid concentration increased due to fertilisation, but differences between KCl and K_2_SO_4_ tubers were not observed Gerendás et al. (2007) and Muttucumaru et al. (2013) confirmed higher free amino acid concentrations due to fertilisation, especially N supply. Furthermore, a relationship between K and N fertilisation is described by the beneficial effects of K application, thus enhancing N uptake and therefore up-regulating amino acid and protein synthesis (Ali et al., 2021). This could explain the higher amino acid and protein concentrations in fertilised tubers in this study. Moreover, additional S application has an impact on amino acid formation and protein synthesis by improving K and P absorption, further supporting the quality-promoting effects of both macroelements (Klikocka et al., 2015; Naumann et al., 2020).

After harvest, resistance to mechanical influences on tubers was analysed *via* thumbnail crack occurrence and skin fracturability measurements. Neither parameter was influenced by fertilisation in either cultivar. With regard to skin fracturability, no year effect was observed, but comparing the two cultivars, the force needed to break the skin was slightly higher in Laura, revealing cultivar-specific properties. Cultivar effects were also observed by Koch et al. (2019b), in which a significantly higher force was needed to fracture the skin of “Omega” compared to Laura. In the study of Koch et al. (2019b), Omega showed a higher tuber DM, which seems to influence tuber fracturability. Similar effects were found in our study comparing Laura and Marabel, since DM and starch content were higher in Laura. No cultivar effect was observed for the thumbnails. However, the thumbnail score of Marabel in 2019 averaged eight, indicating few thumbnail cracks and were therefore twice as high as in 2020 for all the control and fertilisation forms, revealing an effect of cultivation year. This could be related to a different growing season length, as the tubers in 2019 were harvested 2 weeks earlier than in 2020, presuming differences in skin firmness.

With regard to the previous results, it was shown that tuber quality was not significantly affected by KCl compared to K_2_SO_4_ fertilisation. Significant differences between the fertilisation forms could only be found for ascorbic acid. Thus, hypothesis I, KCl supply has adverse effects on tuber quality traits compared to K_2_SO_4_ fertilisation, can be confirmed only to a certain extent.

### Volatile Compounds of Raw Stored and Boiled Tubers Affected by Fertilisation Form

The flavor of potatoes is determined by the interaction of the components taste, texture, and aroma (Jansky, 2010). The aroma originates from volatile compounds, which are synthesised by metabolic processes within the plant and modified by further processing steps, such as boiling or baking (Dresow and Böhm, 2009). These compounds are mainly cultivar-specific, but factors including fertilisation, storage conditions, and processing type (e.g., boiling and baking) have an effect on the formation of volatiles. Although it has been described that storage or fertilisation can have an influence on potatoes’ nutritional content, there are still few publications on these two factors in relation to volatiles determining the aroma profile. Therefore, in this study, the focus was on both the storage process and fertilisation form, as well as on the processing of potatoes by boiling, to determine changes in volatile compounds.

Such thermal processing, in particular, has a major influence on potato aroma and enhances the amount of volatiles compared to raw potatoes (Taylor et al., 2007; Belitz et al., 2008). Thermally driven reactions, such as the Maillard reaction, Strecker degradation, and fatty acid degradation by enzymes, significantly determine potato flavour (Taylor et al., 2007). Most of the detected volatile compounds in this study are potential contributors to potato off-flavours. Pentanal, hexanal, and heptanal increased strongly in both cultivars. In Laura, fertilisation increased these three volatiles compared to the control. Comparing K_2_SO_4_ and KCl supplies, significantly more pentanal and hexanal were found after boiling in KCl tubers, presumably, precursors from lipid degradation of both are enhanced due to Cl^−^. In Marabel, all three volatiles increased after boiling even though in the control, but more pentanal, hexanal, and heptanal were found in K_2_SO_4_ tubers. Pentanal and hexanal are lipid-derived off-flavour compounds in raw and boiled tubers (Duckham et al., 2001; Petersen et al., 2003). It was shown that volatile compounds contributing to off-flavour are enhanced by fertilisation. Moreover, fertilisation effects seem to be cultivar- dependent, since volatiles increased only in tubers of Laura independent from the fertilisation form. Cultivar effects further became evident with significant more increases in volatile compounds formed in Marabel after boiling. These include, in addition to pentanal, hexanal, and heptanal, 2-methylbutanol, octenal, heptenal, nonanal, octenal, 2,4-heptadienal, decanal, 2,4-nonadienal, and 2,4-decadienal. However, high levels of individual volatile components are not always desirable since many products of lipid oxidation are significantly involved in the off-flavour of boiled potatoes (Jansky, 2010). In contrast, volatile substances, which contribute more to the typical potato flavour, are often quantitatively less present but have a higher odour activity value (Jansky, 2010). These include methional, which is often reported as a key contributor to the typical boiled potato aroma (Petersen et al., 1999; Ulrich et al., 2000), and is formed by the Strecker degradation. However, the concentration of methional varies strongly between processing procedures, cultivars, and environmental conditions (Duckham et al., 2002; Oruna-Concha et al., 2002). Duckham et al. (2001) detected this volatile in only five of 11 analysed cultivars. In the present study, methional decreased in both cultivars after storage, more so in fertilised tubers, and could not be detected after boiling in Laura. This reflects findings from Duckham et al. (2001) and shows cultivar-specific effects on methional formation, as well as the fact that potato aroma-specific volatiles do not have to be present at high levels to have an impact on flavour. In a study by Thybo et al. (2006), the aroma composition of cooked tubers of different cultivars, including Marabel, were analysed. The intensity of methional was lower in Marabel than in other cultivars, highlighting cultivar effects. Cultivars used in this study might therefore perform less in terms of methional, explaining the low levels of this volatile compound. Moreover, methional is heat labile and might have been lost during boiling, decomposing to methanethiol, which is further oxidized to dimethyl disulfide (Di et al., 2003). This could explain the low detectable levels of methional after boiling. Moreover, losses may also have occurred due to cutting of tuber into slices, causing some methional to volatilise during the boiling process. Since methional derives from methionine, which contains sulphur, additional sulphur applications could induce differences in methional levels (Duckham et al., 2002). Therefore, K_2_SO_4_ fertilisation might influence by its sulphur component methional levels. Evidence may be provided by comparing the fertilised tubers with the tubers of the control. The level of methional increased more significantly in boiled K_2_SO_4_ tubers of Marabel than in KCl tubers, probably enhancing cultivar specific boiled potato aroma due to K_2_SO_4_ fertilisation. However, this was not observed in this study but could be detected within other cultivars. Studies on the influence of fertilisation on volatiles are scarce. There is little detailed information on the effects of K fertilisation on potato flavour. Descriptions include only possible influences of K on umami intensity or on individual flavour components but without providing more detailed explanations. In addition, further studies are necessary to relate the analysed volatile compounds to sensory perceptions to draw conclusions on potato aroma influenced by fertilisation forms.

After 5 months of storage at 6°C, volatile compounds in raw tubers increased to a lesser extent compared to after boiling. The main source of volatiles in raw potatoes is lipid oxidation products of unsaturated fatty acids, as the content of lipoxygenase is relatively high (Dresow and Böhm, 2009). Especially when the tubers are damaged, e.g., cut for analysis, oxidative and enzymatic processes of the stored lipids are induced (Jansky, 2010), increasing the content of saturated and unsaturated aldehydes and alcohols, which have a low odour threshold but presumably a disadvantageous influence on flavour (Dresow and Böhm, 2009). In addition to hexanal, these included heptenal and 2,4-heptadienal, both of which showed sharp increases after storage in this study. While heptanal showed a higher increase after storage in K_2_SO_4_ and KCl tubers compared to the control in Laura, the increase in Marabel was also high in the control. Fischer (1991) reported, some effect on hexanal and heptanal in freshly harvested tubers due to increasing K fertilisation, depending on the year. Moreover, the author presumed that volatile compounds were affected by increasing N application. However, in Fischer (1991), the tubers were not subjected to any storage period, presuming stronger storage effects compared to the effects of fertilisation on volatile compounds.

Differences in relation to fertilisation form were observed in the volatiles 2,4-nonadienal and 2,4-decadienal in Laura, which increased by 4.28- and 7.75-fold, respectively, in KCl tubers, while they were slightly decreased in stored tubers after K_2_SO_4_ fertilisation. Both are also characterised as potato off-flavours, described in their odour (Supplementary Table S11) as fatty-soapy/-oily, old, rancid, nutty, almond, marzipan (2,4-nonadienal) and fatty, green, onions, chips, chip fat, hot potato, liquorice, and roasty (2,4-decadiendal; Dresow and Böhm, 2009). Cl^−^ may promote the accumulation of unsaturated fatty acids, which form precursors of the two volatile compounds mentioned above, thus favouring the off-flavour. Further studies on unsaturated fatty acids in relation to the fertilisation form could provide detailed insights and should be considered in future studies. Identifying more volatile compounds could provide additional information on the role of fertilisation form on aroma profile. To the best of our knowledge, no other studies have considered the effect of storage on the volatile compounds of raw potatoes. Previous publications have focused mainly on volatiles in boiled tubers or other potato products subjected to storage conditions (Petersen et al., 2003; Laine et al., 2006). The results reveal that fertilising KCl compared to K_2_SO_4_ has some disadvantageous effects on volatile compounds described as lipid-derived off-flavours, thus influencing potato aroma and therefore confirming hypothesis III, KCl supply has adverse effects on volatile compounds.

### Effect of Fertilisation Form During Storage on Tuber Quality

To ensure the demand for fresh potatoes throughout the year, long-term storage after harvest is necessary (Sonnewald and Sonnewald, 2014). Cool storage temperatures prevent tubers from sprouting or spreading pests and diseases, thus minimising quality losses. In our study, tubers were stored for 5 months at 6°C and 95% humidity, which are typical conditions for storing table potatoes. However, biochemical and metabolic processes continue during storage and influence various quality parameters (Pinhero and Yada, 2016). Storage, e.g., significantly influenced the concentration of ascorbic acid in both years and cultivars, with a reduction between 36% and 72%. These results are in line with several authors reporting decreases in ascorbic acid concentration due to long-term storage of 4–6 months (Dale et al., 2003; Hamouz et al., 2018), attributed to the oxidisation to dehydroascorbic acid (Kaundal et al., 2015). In relation to fertilisation, a greater reduction in ascorbic acid concentration after storage was observed in KCl tubers of Marabel in 2019 and 2020. This was not detected in Laura, which could be explained by cultivar specific responses to the interactions between storage and fertilisation (Supplementary Table S3).

In contrast to ascorbic acid, the concentration of reducing sugars increased strongly during 5 months of storage in both years and cultivars and in tuber parts of all fertilisation treatments. The accumulation of sugars is stimulated by low temperatures as they play a role in osmoregulation and cryoprotection (Zörb et al., 2014) and can lead to so-called cold-induced-sweetening of tubers. This involves the vacuolar enzyme acid invertase converting some of the sucrose into glucose and fructose (Wiberley-Bradford et al., 2014), both of which increased in the present study. It has been shown that concentrations of reducing sugars >100 mg per 100 g fresh weight (Bandana et al., 2016) result in unacceptable browning and, furthermore, a bitter taste of fried potato products (Kumar et al., 2004). However, the cultivars investigated in our study belong to the table potato group therefore, browning by frying and thus the formation of acrylamide are of secondary importance. Nevertheless, table potatoes are also processed into French fries for private household use, so a low sugar content after storage is also desirable from the consumer’s perspective to reduce the formation of acrylamide. In addition to storage temperature, sugar accumulation has also been shown to be cultivar-specific (Kumar et al., 2007), thus considering Laura, compared to Marabel, as a favourable cultivar for fried potato products. With respect to fertilisation, the concentration of reducing sugars after storage was higher in the K fertilised tubers compared to the control. Furthermore, sugars accumulated in KCl-treated tubers in both years and cultivars, indicating interaction effects between storage and fertilisation on reducing sugar concentration. The effects of differing K fertilisation on reducing sugar accumulation during storage have to date not been described. However, further research is needed to elucidate the effect of chloride on reducing sugars during storage with respect to other cultivars.

After storage, sucrose was no longer found to be the most abundant sugar but was converted into the reducing sugars glucose and fructose, both of which increased strongly during storage. A decrease in sucrose concentrations was also reported by Ohara-Takada et al. (2005) after storage. Since starch was not strongly degraded during storage, sucrose concentration did not increase and was instead used to be converted into reducing sugars probably to support cryoprotection of the cells (Zörb et al., 2014).

The concentration of total free amino acids and total protein content decreased after storage in 2020 for both cultivars. Halford et al. (2012) observed an increase in amino acids in the first 4 months of storage. In the following months, there was a decrease in the free amino acid concentration, which corresponded to the storage time in our study. The authors presumed that the decrease in concentrations during prolonged storage could indicate the mobilisation of resources needed by tubers for sprouting. In our study, no sprout inhibitors were applied, and the tuber started to sprout on the bud end after 5 months of storage. Proteins might have been degraded during storage into amino acids, which were further were mobilised with sprouting. Therefore, the assumption of Halford et al. (2012) could explain the decreasing amino acid concentration. Overall, storage itself affected the quality parameters of potato tubers, but fertilisation form did not lead to differences during storage in tuber quality, which led to the rejection of hypothesis IV that the fertilisation form additionally influences quality traits during 5 months of storage.

## Conclusion

Fertilising K_2_O as KCl compared to K_2_SO_4_ revealed reductions in several quality-determining traits. The starch content was reduced by 9%–14%, DM by 13%–16%, and ascorbic acid concentration, especially in Laura, by 37% compared to the control and 15% compared to K_2_SO_4_. Moreover, the concentration of reducing sugars was increased in KCl tubers, especially after 5 months of storage, which can contribute to an elevated potential for acrylamide formation. Furthermore, volatile compounds, including pentanal, hexanal, heptanal, 2,4-nonadienal, and 2,4-decadienal, described as lipid-derived off-flavours, were enhanced after storage and boiling in KCl compared to K_2_SO_4_ tubers, probably resulting in differences in potato aroma. Overall, however, fertilisation effects were minor and often interacted with cultivar, year, and storage effects. In addition, the usage of potato tubers should be considered when applying different K fertilisation forms to achieve the best quality properties. In this study, it was shown that KCl can be suitable as K fertiliser for the investigated table potato cultivars, provided that quality parameters—important for processing potatoes, e.g., reducing sugars,—are not considered. However, since volatile compounds are adversely affected by a KCl application both after harvest and after storage, a K_2_SO_4_ fertilisation should be preferred to avoid negative effects on aroma. Moreover, fertilisation with KCl should be reconsidered especially for processing potatoes and should be investigated with appropriate cultivars in further studies.

## Data Availability Statement

The original contributions presented in the study are included in the article/Supplementary Material, further inquiries can be directed to the corresponding author.

## Author Contributions

LW carried out the experiments, did the data analyses and interpretation, drafted, and revised the manuscript. EP and MN designed the study, drafted, and revised the manuscript. All authors contributed to revise the work, gave the final approval of the version to be published, and agreed on all aspects of the work.

## Funding

This study received funding from K+S Minerals and Agriculture GmbH. The funder was not involved in the study design, collection, analysis, interpretation of data, the writing of this article or the decision to submit it for publication. We acknowledge support by the Open Access Publication Funds of the Göttingen University.

## Conflict of Interest

The authors declare that the research was conducted in the absence of any commercial or financial relationships that could be construed as a potential conflict of interest.

## Publisher’s Note

All claims expressed in this article are solely those of the authors and do not necessarily represent those of their affiliated organizations, or those of the publisher, the editors and the reviewers. Any product that may be evaluated in this article, or claim that may be made by its manufacturer, is not guaranteed or endorsed by the publisher.
