# Supplementary material for: Comparison of the Effects of Potassium Sulphate and Potassium Chloride Fertilisation on Quality Parameters, Including Volatile Compounds, of Potato Tubers After Harvest and Storage
Source: Front Plant Sci. 2022 Jul 11;13:920212. doi: 10.3389/fpls.2022.920212 (PMC9310035; doi:10.3389/fpls.2022.920212)
Supplement: Supplementary file 1 [file Data_Sheet_1.docx]

Supplementary Material

# Supplementary Data


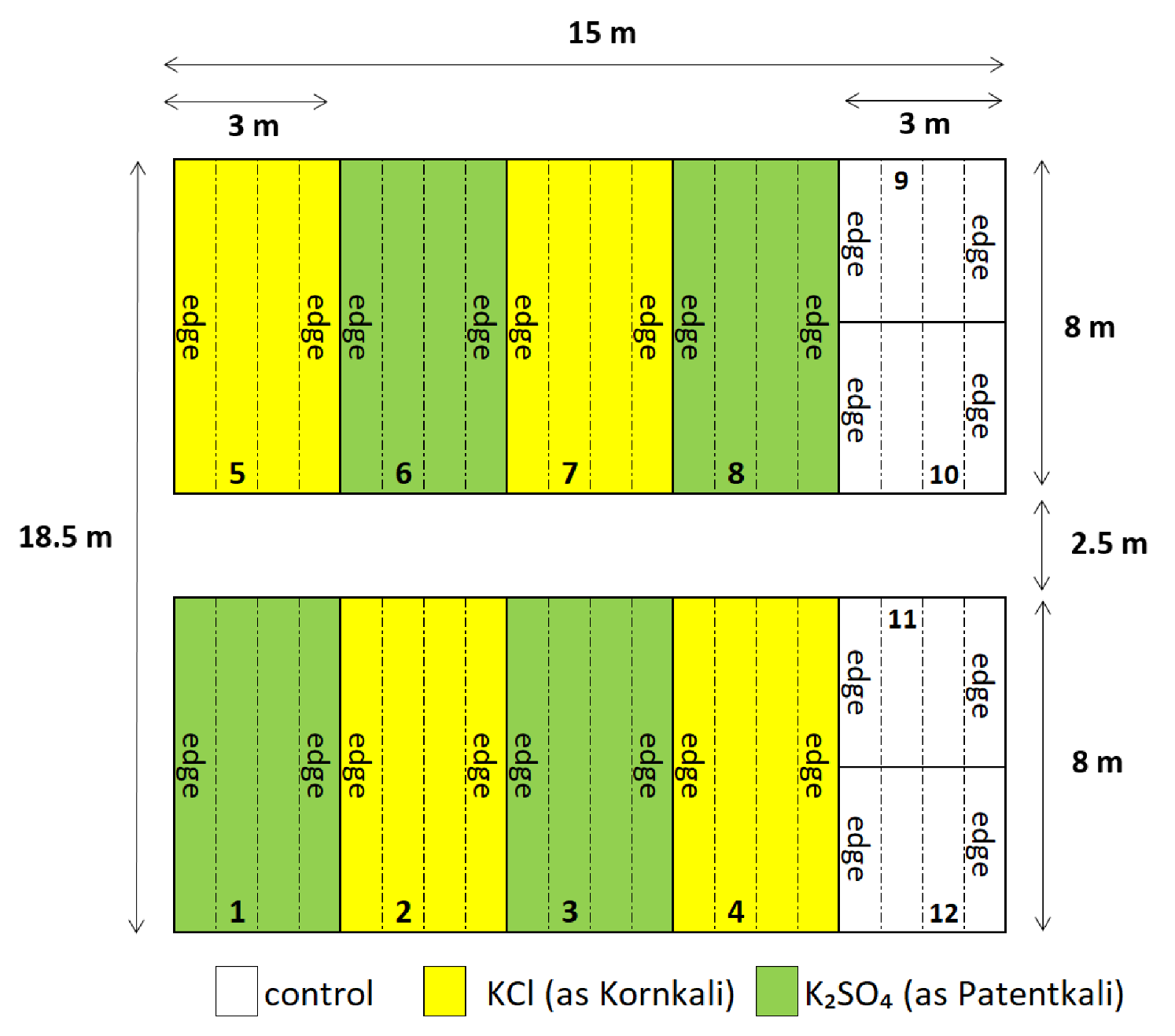


**Figure S1:** Field trial in 2019 with cultivar Marabel.

**Table S1:** Soil nutrient status (mg 100 g^-1^ soil), including N_min_ (kg h^-1^) and characteristics before fertilisation in 2019 and 2020.

| Nutrients | 2019 | 2020 |
| --- | --- | --- |
| K | 20 | 28.1 |
| P | 16 | 8.6 |
| Mg | 9 | 7.6 |
| N_min_ | 72 | 55 |
| Soil type | loamy sand | silty loam |
| pH | 6.7 | 7.1 |

**Table S2:** Nutrient supply (kg h^-1^) in 2019 and 2020.

|  |  | N | P_2_O_5_ | K_2_O | MgO | S |
| --- | --- | --- | --- | --- | --- | --- |
| 2019 | control | 0 | 0 | 0 | 0 | 0 |
|  | K_2_SO_4_ | 88 | 100 | 240 | 80 | 136 |
|  | KCl | 88 | 100 | 240 | 80 | 136 |
| 2020 | control | 0 | 0 | 0 | 0 | 0 |
|  | K_2_SO_4_ | 105 | 100 | 240 | 80 | 136 |
|  | KCl | 105 | 100 | 240 | 80 | 136 |

**Figure S2:** Average day temperature (°C) and precipitation (mm) in Göttingen near to the trial site Reinshof during the vegetation period in 2019.


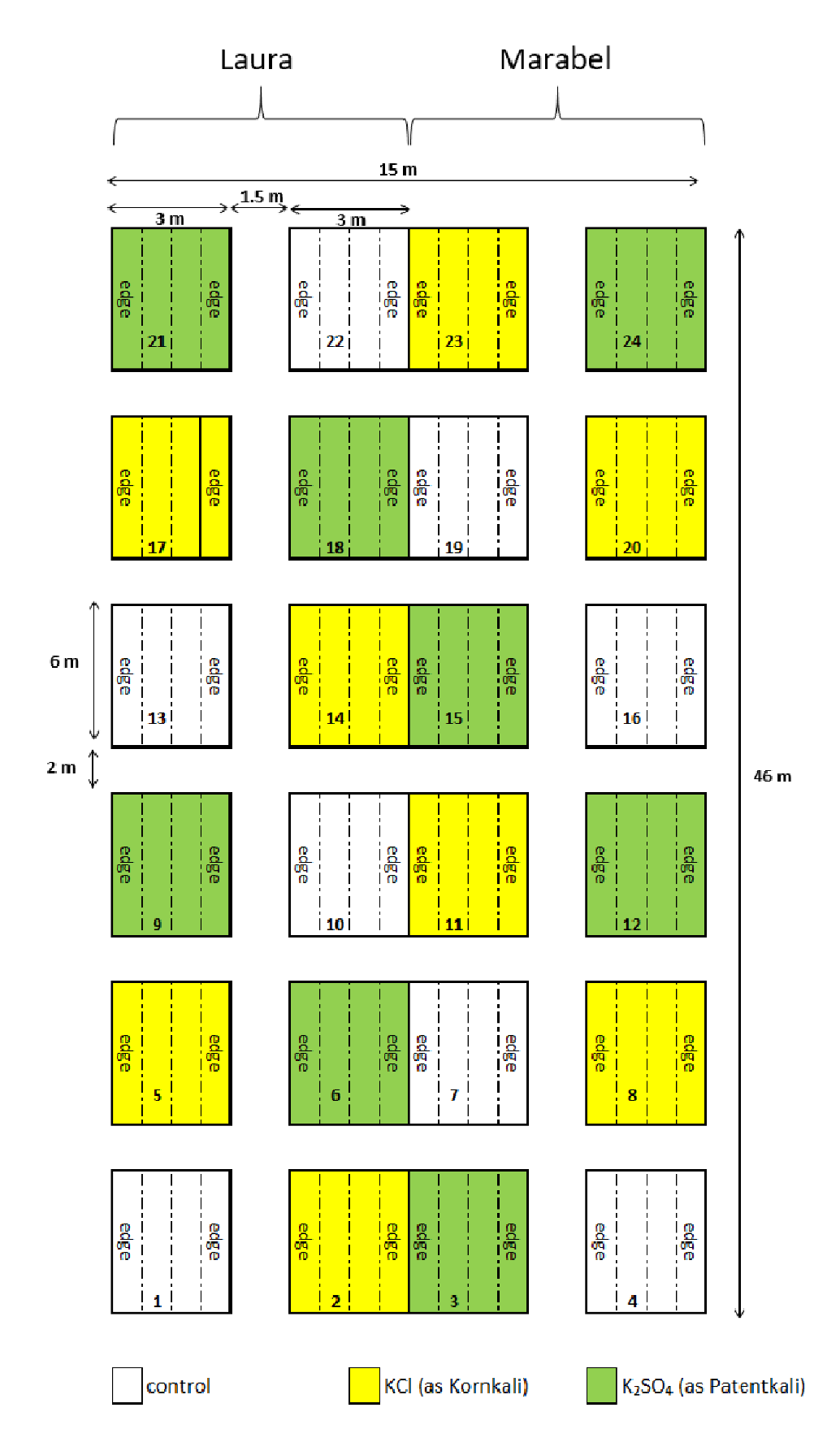


**Figure S3:** Field trial in 2020 with cultivars Marabel and Laura.

**Figure S4:** Average day temperature (°C) and precipitation (mm) in Göttingen near to the trial site Reinshof during the vegetation period in 2020.

**Table S3:** Interaction of factors analysed via analysis of variance (ANOVA) of DM, ascorbic acid (flesh), reducing sugars, and sucrose content in whole tubers.

| Interactions | Dry matter  (%) | Ascorbic acid  (mg g^-1^ DM) | Reducing sugars  (mg g^-1^ DM) | Sucrose  (mg g^-1^ DM) |
| --- | --- | --- | --- | --- |
| Year x Storage | n.s. | ** | *** | *** |
| Cultivar x Storage | n.s. | *** | *** | * |
| Cultivar x Fertilisation | * | n.s. | n.s. | n.s. |
| Storage x Fertilisation | n.s. | * | n.s. | * |

**Table S4:** Interaction of factors analysed via analysis of variance (ANOVA) of chloride sulphate, total free amino acids, and proteins in whole tubers.

| Interactions | K  (mg g^-1^ DM) | Chloride  (mg 100g^-1^ DM) | Sulphate  (mg g^-1^ DM) | Total free amino acids  (µmol g^-1^ DM) | Proteins  (µmol g^-1^ DM) |
| --- | --- | --- | --- | --- | --- |
| Year x Storage | n.s. | *** | *** | *** | ** |
| Year x Fertilisation | ** | n.s. | * | n.s. | n.s. |
| Cultivar x Storage | ** | n.s. | n.s. | n.s. | n.s. |

**Table S5:** Concentration of ascorbic acid (mg g^-1^ DM), reducing sugars (mg g^-1^ DM), and sucrose (mg g^-1^ DM) in tuber parts depending on fertilisation, cultivar, year, and storage.

|  |  |  | Harvest | | | Storage (5 months) | | |
| --- | --- | --- | --- | --- | --- | --- | --- | --- |
| Year | Cultivar | Fertilisation | Stem end | Middle part | Bud end | Stem end | Middle part | Bud end |
| Ascorbic acid | | | | | | | | |
| 2019 | Marabel | control | 1.0±0.05^c^ | 1.4±0.05^b^ | 1.6±0.06^a^ | 0.4±0.04^a^ | 0.5±0.06^a^ | 0.46±0.04^a^ |
|  |  | K_2_SO_4_ | 1.2±0.15^b^ | 1.7±0.18^a^ | 1.8±0.31^a^ | 0.38±0.05^a^ | 0.4±0.05^a^ | 0.43±0.05^a^ |
|  |  | KCl | 1.2±0.10^c^ | 1.7±0.08^b^ | 1.9±0.24^a^ | 0.38±0.03^b^ | 0.44±0.04^a^ | 0.44±0.03^a^ |
| 2020 | Marabel | control | 1.1±0.23^a^ | 1.2±0.14^a^ | 1.7±0.11^a^ | 0.4±0.05^b^ | 0.5±0.06^ab^ | 0.6±0.10^a^ |
|  |  | K_2_SO_4_ | 0.9±0.07^a^ | 1.3±0.31^a^ | 1.5±0.42^a^ | 0.5±0.04^a^ | 0.5±0.06^a^ | 0.5±0.08^a^ |
|  |  | KCl | 0.8±0.11^b^ | 1.2±0.38^ab^ | 1.5±0.19^a^ | 0.4±0.04^a^ | 0.4±.012^a^ | 0.5±0.10^a^ |
|  | Laura | control | n.d. | n.d. | n.d. | n.d. | n.d. | n.d. |
|  |  | K_2_SO_4_ | n.d. | n.d. | n.d. | n.d. | n.d. | n.d. |
|  |  | KCl | n.d. | n.d. | n.d. | n.d. | n.d. | n.d. |
| Reducing sugars | | | | | | | | |
| 2019 | Marabel | control | 23.8±8.3^a^ | 9.7±1.7^b^ | 7.3±1.8^b^ | 39.9±5.1^a^ | 30.7±4.0^b^ | 22.6±3.5^b^ |
|  |  | K_2_SO_4_ | 18.3±4.4^a^ | 8.1±3.1^b^ | 7.7±2.4^b^ | 42.5±9.5^a^ | 40.5±2.9^a^ | 28.0±7.4^b^ |
|  |  | KCl | 30.9±6.4^a^ | 15.1±3.3^b^ | 7.3±3.2^c^ | 42.9±6.5^a^ | 42.2±5.0^a^ | 33.1±4.7^b^ |
| 2020 | Marabel | control | 5.2±2.4^a^ | 2.2±0.5^b^ | 3.0±0.8^ab^ | 44.0±2.1^a^ | 43.1±7.6^a^ | 38.4±15.2^a^ |
|  |  | K_2_SO_4_ | 5.3±0.7^a^ | 2.8±0.9^b^ | 3.0±1.1^b^ | 58.8±11.0^a^ | 46.7±12.6^a^ | 36.6±12.5^a^ |
|  |  | KCl | 4.2±1.3^a^ | 2.5±0.4^a^ | 2.7±0.7^a^ | 51.5±7.1^a^ | 41.1±15.7^a^ | 38.5±6.4^a^ |
|  | Laura | control | 3.5±1.7^a^ | 2.2±1.0^a^ | 3.3±2.6^a^ | 30.1±3.0^a^ | 28.9±1.3^ab^ | 23.5±3.7^b^ |
|  |  | K_2_SO_4_ | 3.8±1.8^a^ | 1.6±0.3^a^ | 1.7±0.8^a^ | 33.4±7.5^a^ | 29.5±6.5^a^ | 26.7±12.2^a^ |
|  |  | KCl | 4.3±1.7^a^ | 2.9±1.7^a^ | 2.6±1.6^a^ | 33.8±4.8^a^ | 33.9±5.2^a^ | 32.2±7.3^a^ |
| Sucrose | | | | | | | | |
| 2019 | Marabel | control | 20.7±2.9^a^ | 20.3±2.1^a^ | 21.2±4.2^a^ | 11.9±2.5a | 8.5±1.1a | 9.5±1.3a |
|  |  | K_2_SO_4_ | 29.9±2.9^a^ | 27.8±6.2ab | 23.3±2.3b | 11.3±1.7a | 10.3±4.4a | 9.4±0.9a |
|  |  | KCl | 23.6±3.3a | 22.4±4.2a | 19.9±4.0a | 12.4±1.3a | 10.2±2.1a | 10.4±2.7a |
| 2020 | Marabel | control | 14.3±3.9^a^ | 11.7±1.9^a^ | 11.8±3.1^a^ | 11.7±2.0^a^ | 10.3±1.2^a^ | 11.1±2.5^a^ |
|  |  | K_2_SO_4_ | 17.2±3.4^a^ | 16.2±1.9^a^ | 14.3±3.8^a^ | 11.3±1.5^a^ | 8.8±1.8^a^ | 9.0±1.7^a^ |
|  |  | KCl | 13.1±2.8^a^ | 14.5±3.3^a^ | 15.4±4.1^a^ | 9.7±1.2^a^ | 9.2±1.6^a^ | 10.8±2.5^a^ |
|  | Laura | control | 6.0±1.7^a^ | 7.4±1.8^a^ | 8.1±2.1^a^ | 6.0±1.6^a^ | 6.3±1.2^a^ | 4.8±1.4^a^ |
|  |  | K_2_SO_4_ | 6.5±0.8^a^ | 6.9±1.7^a^ | 7.5±2.4^a^ | 4.5±2.1^a^ | 4.6±1.8^a^ | 4.8±0 |
|  |  | KCl | 6.6±0.6^a^ | 7.7±1.0^a^ | 7.1±0.9^a^ | 4.0±1.3^a^ | 5.4±0.8^a^ | 2.8±0.3^a^ |
| Significances:  Ascorbic acid: Fertilisation***, Year***, Tuber Part***, Storage***  Reducing sugars: Fertilisation**, Year***, Cultivar***, Tuber part***, Storage***  Sucrose: Fertilisation***, Year***, Cultivar***, Tuber part n.s., Storage*** | | | | | | | | |

Mean ± SD (n=4), *, **, and *** for p < 0.05, 0.01 and 0.001, respectively. Small letters = differences between stem end, middle part, and bud end within one parameter and fertilisation treatment after harvest or 5 months of storage. Effect of year considers differences between Marabel in 2019 and 2020, cultivar effects describe differences between Laura and Marabel in 2020, and storage effects refer to data of both cultivars and both years. n.d. = not determined.

**Table S6:** Concentration of chloride (mg g^-1^ DM) and sulphate (mg g^-1^ DM) in tuber skin and flesh depending on fertilisation, cultivar, year and storage.

|  | | | Harvest | | Storage (5 months) | |
| --- | --- | --- | --- | --- | --- | --- |
| Year Cultivar Fertilisation | | | Skin | Flesh | Skin | Flesh |
| Chloride | | | | | | |
| 2019 | Marabel | control | 3.63±0.26^a^ | 2.52±0.19^b^ | 5.29±0.18^a^ | 4.38±0.03^b^ |
|  |  | K_2_SO_4_ | 3.49±0.14^a^ | 2.66±0.18^b^ | 5.41±0.29^a^ | 4.68±0.36^b^ |
|  |  | KCl | 5.53±0.49^a^ | 4.51±0.40^b^ | 7.87±0.66^a^ | 6.24±0.38^b^ |
| 2020 | Marabel | control | 2.26±0.19^a^ | 2.04±0.32^a^ | 4.27±1.08^a^ | 2.84±0.10^a^ |
|  |  | K_2_SO_4_ | 2.75±0.08^a^ | 2.33±0.22^b^ | 4.29±0.37^a^ | 3.16±0.11^b^ |
|  |  | KCl | 4.23±0.78^a^ | 3.31±0.68^a^ | 6.60±0.81^a^ | 5.08±0.25^b^ |
|  | Laura | control | 2.80±0.34^a^ | 1.44±0.33^b^ | 4.91±0.76^a^ | 2.38±0.02^b^ |
|  |  | K_2_SO_4_ | 3.69±0.24^a^ | 1.73±0.27^b^ | 5.30±2.10^a^ | 2.78±0.02^b^ |
|  |  | KCl | 5.44±0.55^a^ | 2.92±0.23^b^ | 9.32±2.39^a^ | 3.59±0.41^b^ |
| Sulphate | | | | | | |
| 2019 | Marabel | control | 0.95±0.03^b^ | 1.58±0.09^a^ | 2.07±0.08^b^ | 2.39±0.08^a^ |
|  |  | K_2_SO_4_ | 1.48±0.10^b^ | 1.95±0.12^a^ | 2.67±0.26^a^ | 3.00±0.63^a^ |
|  |  | KCl | 1.37±0.07^b^ | 1.71±0.12^a^ | 2.55±0.20^a^ | 2.71±0.26^a^ |
| 2020 | Marabel | control | 3.31±0.29^a^ | 2.73±0.38^a^ | 2.92±0.23^a^ | 3.11±0.20^a^ |
|  |  | K_2_SO_4_ | 3.40±0.30^a^ | 2.99±0.08^a^ | 2.98±0.20^a^ | 3.19±0.22^a^ |
|  |  | KCl | 3.24±0.25^a^ | 2.59±0.15^b^ | 2.78±0.11^b^ | 3.02±0.04^a^ |
|  | Laura | control | 3.55±0.25^a^ | 2.80±0.30^b^ | 3.09±0.17^a^ | 3.16±0.16^a^ |
|  |  | K_2_SO_4_ | 4.23±0.50^a^ | 3.39±0.18^a^ | 3.16±0.41^a^ | 3.55±0.17^a^ |
|  |  | KCl | 2.96±0.35^a^ | 2.63±0.39^a^ | 3.05±0.17^a^ | 3.29±0.17^a^ |
| Significances:  Chloride: Fertilisation***, Year***, Cultivar*, Tuber part***, Storage***  Sulphate: Fertilisation***, Year***, Cultivar*, Tuber part n.s., Storage*** | | | | | | |

Mean ± SD (n=4), *, **, and *** for p < 0.05, 0.01 and 0.001, respectively. Small letters = differences between skin and flesh within one parameter and fertilisation treatment after harvest or 5 months of storage. Effect of year considers differences between Marabel in 2019 and 2020 and cultivar effects describe differences between Laura and Marabel in 2020, and storage effects refer to data of both cultivars and both years.

**Table S7:** Distribution of K (mg g^-1^ DM), chloride (mg g^-1^ DM), and sulphate (mg g^-1^ DM) in tuber parts depending on fertilisation, cultivar, year and storage.

|  |  |  | Harvest | | | Storage (5 months) | | |
| --- | --- | --- | --- | --- | --- | --- | --- | --- |
| Year | Cultivar | Fertilisation | Stem end | Middle | Bud end | Stem end | Middle | Bud end |
| Potassium | | | | | | | | |
| 2019 | Marabel | control | 16.62±0.88c | 22.14±0.68b | 26.28±1.37a | 14.45±0.32c | 20.44±0.89b | 24.88±0.91a |
|  |  | K_2_SO_4_ | 20.04±2.78c | 23.21±0.65b | 27.93±1.72a | 18.40±1.39c | 22.69±0.85b | 27.35±1.84a |
|  |  | KCl | 19.92±1.13c | 23.52±0.99b | 27.90±1.63a | 20.14±1.51c | 23.75±1.79b | 26.53±1.51a |
| 2020 | Marabel | control | 15.95±0.61c | 20.53±0.83b | 25.08±1.03a | 15.73±1.30 | 19.62±0.53 | 24.29±1.13 |
|  |  | K_2_SO_4_ | 16.26±0.87c | 20.74±1.15b | 24.83±1.13a | 15.85±0.85 | 20.80±2.59 | 24.06±0.71 |
|  |  | KCl | 17.68±1.16c | 19.15±1.89b | 24.70±0.77a | 16.78±1.26 | 21.39±4.23 | 24.28±1.69 |
|  | Laura | control | 14.54±0.97c | 19.75±0.84b | 24.23±0.51a | 13.90±0.26 | 18.16±0.52 | 22.75±2.44 |
|  |  | K_2_SO_4_ | 15.23±1.14c | 20.42±0.72b | 24.62±0.40a | 14.30±0.67 | 19.21±1.45 | 24.06±1.38 |
|  |  | KCl | 15.95±1.07c | 20.64±0.84b | 25.05±0.30a | 16.41±1.10 | 21.09±0.92 | 25.08±1.62 |
| Chloride | | | | | | | | |
| 2019 | Marabel | control | 2.81±0.17a | 2.69±0.21a | 2.31±0.19b | 4.86±0.29a | 4.67±0.11a | 4.48±0.29a |
|  |  | K_2_SO_4_ | 2.96±0.27a | 2.76±0.17a | 2.36±0.19b | 4.92±0.32a | 4.77±0.27a | 4.61±0.23a |
|  |  | KCl | 6.50±0.51a | 4.39±0.35a | 3.35±0.76b | 7.88±0.49a | 6.49±0.33b | 5.34±0.21c |
| 2020 | Marabel | control | 1.96±0.41a | 2.06±0.25a | 1.85±0.18a | 2.68±0.16a | 2.87±0.13a | 2.77±0.07a |
|  |  | K_2_SO_4_ | 2.48±0.23ab | 2.49±0.22a | 2.05±0.21b | 3.35±0.15a | 3.36±0.14a | 3:06±0.17a |
|  |  | KCl | 5.27±0.70a | 3.82±0.27b | 2.64±0.21c | 6.04±0.74a | 4.37±0.68b | 3.63±0.19b |
|  | Laura | control | 1.60±0.17a | 1.88±0.32a | 2.12±0.40a | 2.41±0.22b | 2.58±0.14ab | 2.91±0.24a |
|  |  | K_2_SO_4_ | 1.60±0.11b | 1.95±0.29ab | 2.21±0.18a | 2.63±0.13c | 2.87±0.13b | 3.33±0.10a |
|  |  | KCl | 3.44±0.51a | 3.43±0.26a | 3.02±0.27a | 4.09±0.39a | 4.19±0.36a | 4.14±0.23a |
| Sulphate | | | | | | | | |
| 2019 | Marabel | control | 1.78±0.08a | 1.52±0.08b | 1.22±0.07c | 2.58±0.08a | 2.35±0.07b | 2.24±0.07b |
|  |  | K_2_SO_4_ | 2.25±0.10a | 1.98±0.14b | 1.67±0.10c | 3.21±0.40a | 2.91±0.25ab | 2.74±0.25b |
|  |  | KCl | 2.02±0.16a | 1.71±0.15b | 1.52±0.14c | 3.02±0.58a | 2.84±0.50a | 2.57±0.23a |
| 2020 | Marabel | control | 3.42±0.12a | 2.80±0.16b | 2.44±0.30b | 3.35±0.20a | 3.03±0.16a | 2.63±0.13b |
|  |  | K_2_SO_4_ | 3.52±0.28a | 2.96±0.29ab | 2.54±0.31b | 3.60±0.14a | 3.19±0.14b | 2.80±0.14c |
|  |  | KCl | 3.14±0.34a | 2.64±0.20b | 2.31±0.14b | 3.35±0.17a | 2.89±0.25b | 2.62±0.22b |
|  | Laura | control | 3.58±0 21a | 2.57±0.25b | 2.00±0.39b | 3.69±0.37a | 3.12±0.20ab | 2.54±0.47b |
|  |  | K_2_SO_4_ | 4.22±0.18a | 3.08±0.39b | 2.41±0.24c | 4.09±0.15a | 3.31±0.12b | 2.77±0.13c |
|  |  | KCl | 4.33±0.24a | 3.06±0.20b | 2.35±0.12c | 3.83±0.43a | 3.21±0.15b | 2.71±0.08b |
| Significances:  Potassium: Fertilisation n.s., Year***, Cultivar n.s., Tuber part***, Storage n.s.  Chloride: Fertilisation***, Year**, Cultivar n.s., Tuber part**, Storage***  Sulphate: Fertilisation n.s., Year***, Cultivar n.s., Tuber part***, Storage** | | | | | | | | |

Mean ± SD (n=4), *, **, and *** for p < 0.05, 0.01 and 0.001, respectively. Small letters = differences between stem end, middle part, and bud end within one parameter and fertilisation treatment after harvest or 5 months of storage. Effect of year considers differences between Marabel in 2019 and 2020, cultivar effects describe differences between Laura and Marabel in 2020, and storage effects refer to data of both cultivars and both years.

**Table S8:** Distribution of K (mg g^-1^ DM), dry matter (%), ascorbic acid (mg g^-1^ DM), reducing sugars (mg g^-1^ DM), and sucrose (mg g^-1^ DM) in tuber skin and flesh depending on fertilisation, cultivar, year and storage.

|  | | | Harvest | | Storage (5 months) | |
| --- | --- | --- | --- | --- | --- | --- |
| Year | Cultivar | Fertilisation | Skin | Flesh | Skin | Flesh |
| Potassium | | | | | | |
| 2019 | Marabel | control | 30.37±0.56^a^ | 20.15±0.31^b^ | 27.30±1.31^a^ | 18.97±1.23^b^ |
|  |  | K_2_SO_4_ | 29.91±1.82^a^ | 20.91±1.39^b^ | 28.83±2.21^a^ | 20.65±0.65^b^ |
|  |  | KCl | 30.42±1.39^a^ | 24.09±3.22^b^ | 32.52±2.01^a^ | 22.05±1.07^b^ |
| 2020 | Marabel | control | 25.43±0.55^a^ | 20.28±1.11^b^ | 26.40±0.65^a^ | 18.02±0.83^b^ |
|  |  | K_2_SO_4_ | 24.94±1.33^a^ | 20.50±1.98^b^ | 26.81±2.45^a^ | 17.96±0.71^b^ |
|  |  | KCl | 25.15±0.74^a^ | 20.70±1.51^b^ | 28.06±4.35^a^ | 20.52±1.07^b^ |
|  | Laura | control | 26.71±2.43^a^ | 18.56±0.20^b^ | 29.77±3.69^a^ | 17-54±0.63^b^ |
|  |  | K_2_SO_4_ | 27.93±1.16^a^ | 19.75±1.25^b^ | 28.23±5.37^a^ | 18.52±0.39^b^ |
|  |  | KCl | 24.98±0.89^a^ | 21.48±1.94^b^ | 29.77±4.43^a^ | 19.41±1.30^b^ |
| Dry matter | | | | | | |
| 2019 | Marabel | control | 20.13±0.57^a^ | 23.71±0.56^b^ | 21.26±0.49^a^ | 23.81±0.51^b^ |
|  |  | K_2_SO_4_ | 18.56±0.93^a^ | 21.49±0.86^b^ | 19.65±0.57^a^ | 21.76±0.78^b^ |
|  |  | KCl | 18.40±1.14^a^ | 20.32±1.30^b^ | 17.40±0.81^a^ | 19.54±1.11^b^ |
| 2020 | Marabel | control | 17.97±0.56^a^ | 21.74±0.87^b^ | 19.57±0.70^a^ | 22.28±0.37^b^ |
|  |  | K_2_SO_4_ | 17.93±0.68^a^ | 21.12±1.11^b^ | 18.80±0.72^a^ | 20.05±1.35^b^ |
|  |  | KCl | 17.18±0.95^a^ | 20.32±1.16^b^ | 18.37±0.70^a^ | 19.41±1.51^b^ |
|  | Laura | control | 17.60±0.63^a^ | 24.56±1.51^b^ | 20.22±1.25^a^ | 24.42±0.57^b^ |
|  |  | K_2_SO_4_ | 16.26±0.97^a^ | 22.76±0.86^b^ | 18.85±0.40^a^ | 21.99±1.01^b^ |
|  |  | KCl | 15.62±0.91^a^ | 21.79±1.49^b^ | 18.36±0.44^a^ | 21.85±0.58^b^ |
| Ascorbic acid | | | | | | |
| 2019 | Marabel | control | 0.64±0.13^a^ | 1.66±0.18^b^ | 0.29±0.04^a^ | 0.56±0.02^b^ |
|  |  | K_2_SO_4_ | 0.73±0.13^a^ | 1.76±0.22^b^ | 0.28±0.08^a^ | 0.54±0.04^b^ |
|  |  | KCl | 0.65±0.15^a^ | 1.80±0.11 ^b^ | 0.28±0.04^a^ | 0.51±0.07^b^ |
| 2020 | Marabel | control | 0.86±0.06^a^ | 1.99±0.09^b^ | 0.38±0.02^a^ | 0.81±.013^b^ |
|  |  | K_2_SO_4_ | 0.61±0.11^a^ | 1.64±0.13^b^ | 0.34±0.07^a^ | 0.85±0.13^b^ |
|  |  | KCl | 0.63±0.11^a^ | 1.69±0.18^b^ | 0.26±0.05^a^ | 0.66±0.13^b^ |
|  | Laura | control | n.d. | 1.17±0.13^b^ | n.d. | 0.36±0.06^b^ |
|  |  | K_2_SO_4_ | n.d. | 1.01±0.14^b^ | n.d. | 0.34±0.05^b^ |
|  |  | KCl | n.d. | 0.84±0.12^b^ | n.d. | 0.36±0.04^b^ |
| Reducing sugars | | | | | | |
| 2019 | Marabel | control | 25.64±4.12^a^ | 10.36±2.42^b^ | 18.52±2.11^a^ | 37.39±7.24^b^ |
|  |  | K_2_SO_4_ | 17.19±5.10^a^ | 8.23±2.99^b^ | 16.36±3.77^a^ | 41.04±6.60^b^ |
|  |  | KCl | 27.90±6.60^a^ | 12.01±4.36^b^ | 20.85±6.41^a^ | 44.54±10.09^b^ |
| 2020 | Marabel | control | 5.76±1.80^a^ | 2.90±0.66^b^ | 20.57±5.53^a^ | 47.57±5.87^b^ |
|  |  | K_2_SO_4_ | 4.60±0.38^a^ | 2.93±1.09^b^ | 24.11±8.88^a^ | 48.66±10.30^b^ |
|  |  | KCl | 6.13±2.65^a^ | 2.76±1.35^b^ | 23.60±6.15^a^ | 49.04±4.11^b^ |
| Table S8 continued | | |  |  |  |  |
|  | Laura | control | 6-26±3.09^a^ | 2.13±1.02^b^ | 16.69±8.04^a^ | 29.57±5.77^b^ |
|  |  | K_2_SO_4_ | 5.30±0.40^a^ | 2.27±0.83^b^ | 16.24±2.84^a^ | 32.44±7.24^b^ |
|  |  | KCl | 3.90±0.96^a^ | 2.37±1.80^b^ | 14.54±3.92^a^ | 33.94±9.12^b^ |
| Sucrose | | | | | | |
| 2019 | Marabel | control | 12.35±2.40^a^ | 23.65±7.40^b^ | 8.73±0.90^a^ | 10.56±1.54 ^b^ |
|  |  | K_2_SO_4_ | 13.39±2.89^a^ | 29.02±5.11^b^ | 9.23±0.80^a^ | 10.69±1.65^b^ |
|  |  | KCl | 13.33±2.06^a^ | 23.02±4.74^b^ | 9.16±1.64^a^ | 10.06±1.34^b^ |
| 2020 | Marabel | control | 5.86±1.75^a^ | 15.75±2.24^b^ | 6.79±4.43^a^ | 10.22±1.70^b^ |
|  |  | K_2_SO_4_ | 6.83±1.18^a^ | 14.46±3.68^b^ | 7.75±2.54^a^ | 7.55±0.82^b^ |
|  |  | KCl | 6.17±1.92^a^ | 19.05±4.28^b^ | 9.26±2.18^a^ | 8.67±0.81^b^ |
|  | Laura | control | 3.57±1.95^a^ | 7.79±1.66^b^ | 3.71±2.58^a^ | 7.81±2.82^b^ |
|  |  | K_2_SO_4_ | 3.40±1.22^a^ | 7.66±1.65^b^ | 5.76 | 5.40±2.07^b^ |
|  |  | KCl | 4.70±1.59^a^ | 7.06±2.77^b^ | n.d. | 7.67±1.49^b^ |
| Significances  Potassium: Fertilisation n.s., Year**, Cultivar n.s., Tuber part***, Storage n.s.  DM: Fertilisation*, Cultivar n.s., Year n.s., Tuber part***, Storage n.s.  Ascorbic acid: Fertilisation n.s., Year n.s., Cultivar***, Tuber part***, Storage***  Reducing sugars: Fertilisation n.s., Year***, Cultivar n.s., Tuber part***, Storage***  Sucrose: Fertilisation n.s., Year***, Cultivar***, Tuber part***, Storage*** | | | | | | |

Mean ± SD (n=4), *, **, and *** for p < 0.05, 0.01 and 0.001, respectively. Small letters = differences between skin and flesh within one parameter and fertilisation treatment after harvest or 5 months of storage. Effect of year considers differences between Marabel in 2019 and 2020, cultivar effects describe differences between Laura and Marabel in 2020, and storage effects refer to data of both cultivars and both years. n.d. = not determined.


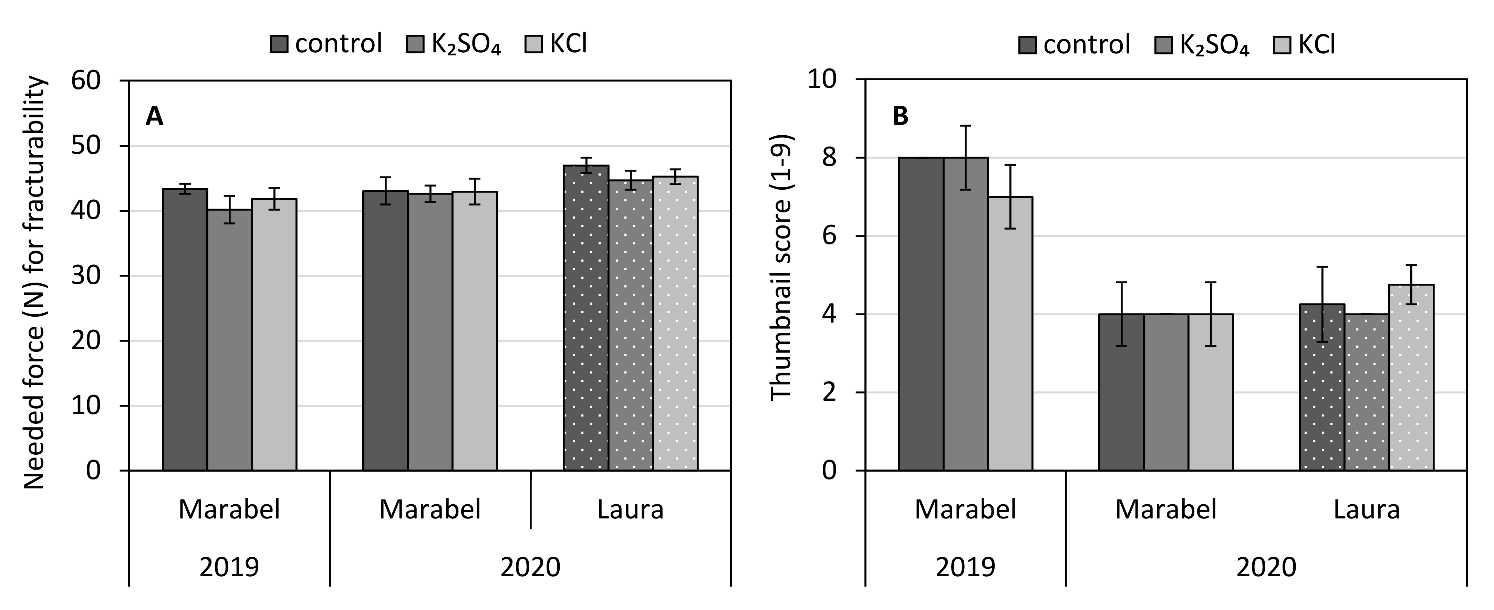


**Figure S5:** Needed force for skin fracturability and thumbnail score of tubers depending on fertilisation, year and cultivar. Mean ± SD (n = 4). *, **, and *** for p < 0.05, 0.01 and 0.001, respectively. n.s. = not significant. Effect of year considers differences of tubers after harvest between 2019 and 2020 for Marabel and cultivar effects describe differences between Laura and Marabel in 2020. Skin fracturability: Fertilisation n.s., Year n.s., Cultivar*; Thumbnail score: Fertilisation n.s., Year***, Cultivar n.s.


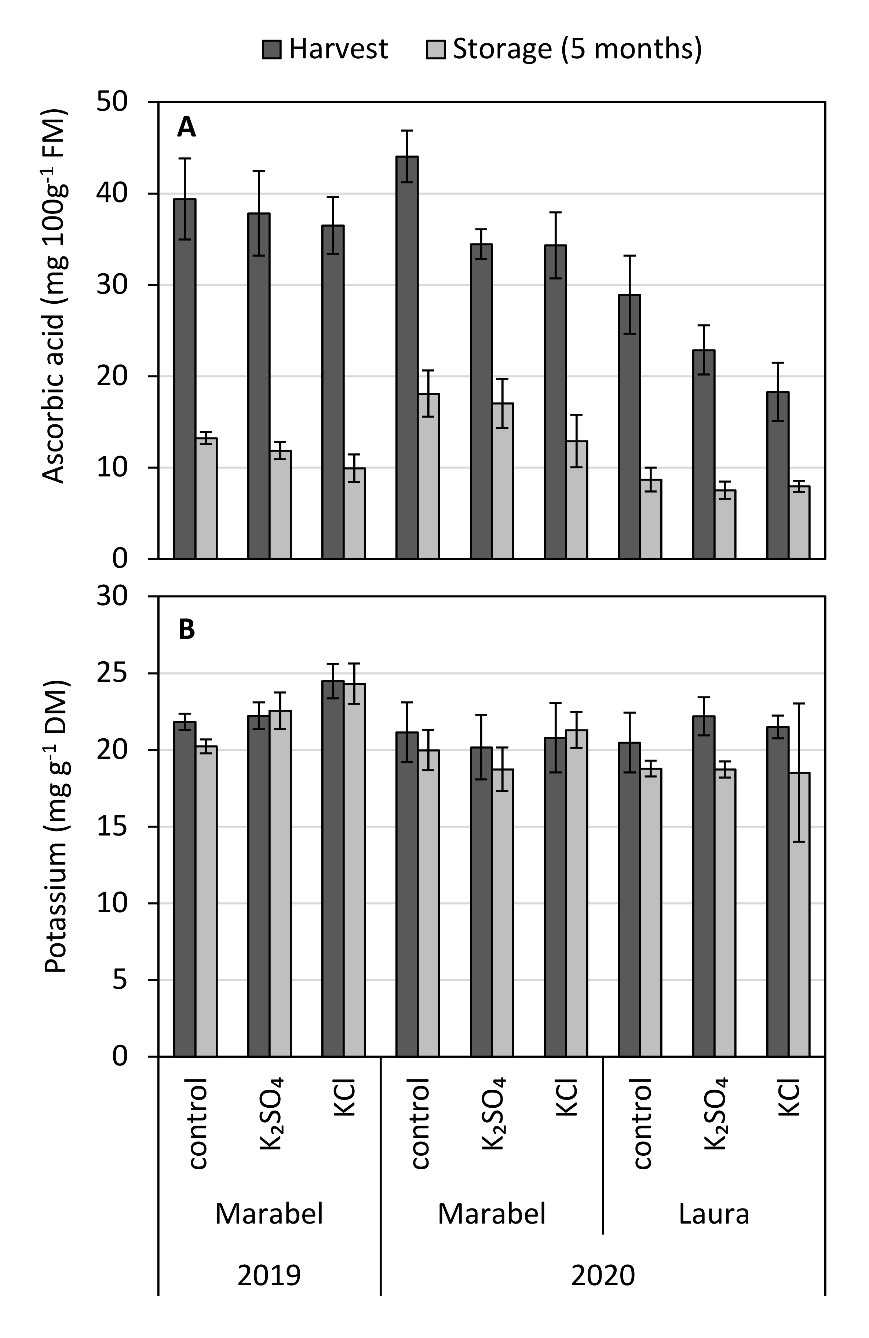


**Figure S6:** Ascorbic acid (A) and K (B) concentrations in tubers depending on fertilisation, cultivar, year, and storage. Mean ± SD (n = 4). *, **, and *** for p < 0.05, 0.01 and 0.001, respectively. n.s. = not significant. Effect of year considers differences of tubers after harvest between 2019 and 2020 for Marabel, cultivar effects describe differences between Laura and Marabel in 2020, and storage effects refer to data of both cultivars and both years. Ascorbic acid: Fertilisation**, Year*; Cultivar***; Storage***; Potassium: Fertilisation ***, Year***, Cultivar n.s., Storage*.

**Table S9:** Fold changes of volatile compounds comparing raw KCl and K_2_SO_4_ tubers to raw unfertilised control tubers at harvest and after 5 months of storage at 6°C in 2020 in cultivar Laura and Marabel.

|  | **Laura** | | | | **Marabel** | | | |
| --- | --- | --- | --- | --- | --- | --- | --- | --- |
|  | **Harvest** | | **Storage** | | **Harvest** | | **Storage** | |
| **Volatile compound** | **KCl** | **K₂SO₄** | **KCl** | **K₂SO₄** | **KCl** | **K₂SO₄** | **KCl** | **K₂SO₄** |
| Pentanal | 0.09 | 0.30 | 1.22 | 0.67 | 0.80 | 0.20 | 0.00 | 0.00 |
| Hexanal | 0.09 | 0.21 | 0.67 | 0.18 | 2.01 | 1.01 | 0.49 | 1.40 |
| Heptanal | 0.23 | 0.30 | 0.69 | 1.11 | 1.40 | 1.00 | 1.40 | 1.15 |
| 2-Methylbutanol | 0.95 | 0.75 | 1.83 | 1.13 | 0.87 | 1.03 | 0.98 | 1.05 |
| 3-Methylbutanol | 0.86 | 0.96 | 0.61 | 1.45 | 1.06 | 1.23 | 1.05 | 1.26 |
| 2-Pentylfuran | 0.57 | 0.82 | 0.74 | 0.60 | 1.26 | 1.08 | 0.60 | 0.98 |
| 1-Pentanol | 0.60 | 0.90 | 0.91 | 0.78 | 1.34 | 1.03 | 0.75 | 0.90 |
| Octenal | 0.59 | 0.86 | 0.99 | 0.84 | 0.75 | 0.91 | 0.88 | 0.93 |
| 2-Heptenal | 0.34 | 0.43 | 0.73 | 0.61 | 1.37 | 1.11 | 0.91 | 1.14 |
| Nonanal | 0.18 | 0.14 | 1.00 | 1.13 | 0.86 | 1.57 | 1.14 | 1.14 |
| 2-Octenal | 0.69 | 1.10 | 0.81 | 0.68 | 1.14 | 1.01 | 0.69 | 0.97 |
| 2-Isopropyl-3-methoxy pyrazine | 0.72 | 0.88 | 0.81 | 0.60 | 1.01 | 1.08 | 0.64 | 0.99 |
| 1-Octen-3-ol | 0.72 | 0.94 | 0.73 | 0.81 | 1.13 | 0.88 | 0.87 | 0.87 |
| Methional | 1.28 | 1.28 | 0.32 | 0.36 | 1.22 | 1.04 | 1.00 | 1.00 |
| 2,4-Heptadienal | 2.13 | 1.69 | 1.06 | 1.14 | 1.26 | 0.69 | 1.02 | 0.97 |
| Decanal | 0.41 | 0.69 | 1.02 | 0.52 | 1.17 | 0.83 | 0.34 | 0.97 |
| 2-Nonenal | 1.24 | 1.11 | 0.34 | 0.57 | 0.90 | 0.89 | 0.84 | 0.80 |
| 2,4-Nonadienal | 0.17 | 0.41 | 0.71 | 0.27 | 2.31 | 1.23 | 0.13 | 0.83 |
| 2,4-Decadienal | 0.17 | 0.54 | 0.74 | 0.23 | 1.86 | 1.35 | 0.10 | 0.67 |

Values are expressed as relative fold changes comparing mean values of raw control tubers with mean values of raw KCl or K_2_SO_4_ tubers at harvest or after 5 months of storage. Colour scale showing increase (>1 = red) or decrease (<1 = blue) of individual volatile compounds in KCl and K_2_SO_4_ tubers. n = 4.

**Table S10:** Fold changes of volatile compounds comparing boiled KCl and K_2_SO_4_ tubers to boiled unfertilised control tubers at harvest and after 5 months of storage at 6°C in 2020 in cultivar Laura and Marabel.

|  | **Laura** | | | | **Marabel** | | | |
| --- | --- | --- | --- | --- | --- | --- | --- | --- |
|  | **Harvest** | | **Storage** | | **Harvest** | | **Storage** | |
| **Volatile compound** | **KCl** | **K₂SO₄** | **KCl** | **K₂SO₄** | **KCl** | **K₂SO₄** | **KCl** | **K₂SO₄** |
| Pentanal | 0.95 | 1.74 | 1.56 | 1.29 | 1.06 | 0.87 | 0.72 | 0.92 |
| Hexanal | 1.12 | 0.90 | 2.47 | 1.63 | 1.05 | 0.98 | 0.66 | 0.90 |
| Heptanal | 0.90 | 1.39 | 0.92 | 1.28 | 0.98 | 0.94 | 0.97 | 0.98 |
| 2-Methylbutanol | 0.99 | 0.74 | 1.75 | 1.25 | 0.73 | 0.89 | 0.67 | 0.94 |
| 3-Methylbutanol | 2.00 | 1.88 | 0.90 | 0.75 | 1.17 | 1.05 | 0.75 | 0.80 |
| 2-Pentylfuran | 4.19 | 2.07 | 1.39 | 0.95 | 1.05 | 1.19 | 0.79 | 1.02 |
| 1-Pentanol | 1.25 | 0.94 | 1.69 | 1.50 | 1.36 | 0.78 | 0.67 | 0.92 |
| Octenal | 0.57 | 0.54 | 1.25 | 1.25 | 0.93 | 0.77 | 0.48 | 0.68 |
| 2-Heptenal | 0.96 | 0.83 | 1.18 | 0.86 | 1.01 | 0.84 | 0.59 | 0.84 |
| Nonanal | 1.14 | 1.29 | 1.28 | 1.05 | 0.87 | 4.70 | 0.80 | 1.10 |
| 2-Octenal | 1.08 | 0.87 | 1.80 | 1.20 | 1.17 | 1.13 | 0.76 | 1.01 |
| 2-Isopropyl-3-methoxy pyrazine | 0.89 | 0.30 | 1.73 | 0.90 | 0.62 | 6.25 | 0.70 | 0.70 |
| 1-Octen-3-ol | 1.11 | 0.72 | 1.50 | 1.30 | 1.10 | 1.06 | 0.76 | 0.98 |
| Methional | 0.00 | 0.00 | 1.50 | 1.31 | 4.00 | 10.67 | 0.90 | 0.90 |
| 2,4-Heptadienal | 0.55 | 1.54 | 1.30 | 1.25 | 1.18 | 1.12 | 0.99 | 0.83 |
| Decanal | 1.05 | 0.70 | 1.32 | 1.00 | 1.07 | 0.95 | 1.25 | 1.14 |
| 2-Nonenal | 1.06 | 1.20 | 0.71 | 0.71 | 0.77 | 0.85 | 0.69 | 0.89 |
| 2,4-Nonadienal | 1.13 | 0.84 | 2.45 | 1.70 | 1.15 | 1.18 | 0.70 | 0.93 |
| 2,4-Decadienal | 1.12 | 0.77 | 1.58 | 1.13 | 1.19 | 1.16 | 0.67 | 0.94 |

Values are expressed as relative fold changes comparing mean values of boiled control tubers with mean values of boiled KCl or K_2_SO_4_ tubers at harvest or after 5 months of storage. Colour scale showing increase (>1 = red) or decrease (<1 = blue) of individual volatile compounds in KCl and K_2_SO_4_ tubers. n = 4.

**Table S11:** Odour description of aroma compounds.

| Compound | Raw | Boiled | Odour description | Origin | References |
| --- | --- | --- | --- | --- | --- |
| Pentanal | x |  | green, almond, marzipan | Lipid degradation | Petersen et al. (1999)  Khan et al. (1977) |
| Hexanal | x | x | green, grass | Lipid degradation | Petersen et al. (1999) |
| Heptanal | x | x | soapy-fruity, resinous | Lipid degradation | Khan et al. (1977) |
| 2-Methylbutanol | x | x | malty | Sugar degradation | Duckham et al. (2002) |
| 3-Methylbutanol | x | x | malty | Sugar degradation | Duckham et al. (2002) |
| 2-Pentylfuran | x | x | unpleasant, green, beany, grassy, cooked | Lipid degradation | Oruna-Concha et al. (2001)  Duckham et al. (2002) |
| 1-Pentanol | x | x | malty | Lipid degradation | Petersen et al. (1999) |
| Octenal |  | x |  | Lipid degradation | Oruna-Concha et al. (2001) |
| Heptenal |  | x | cardboard-like | Lipid degradation | Duckham et al. (2001) |
| Nonanal |  | x | rancid, boiled potato | Lipid degradation | Petersen et al. (1999) |
| 2-Octenal | x | x | baked potato, boiled, chips | Lipid degradation | Petersen et al. (1999) |
| 2-Isopropyl-3-methoxypyrazin | x | x | Earthy, green, peas, raw potato | Methoxypyrazines | Duckham et al. (2001)  Petersen et al. (1999) |
| 1-Octen-3-ol | x | x | earthy, mushroom | Lipid degradation | Oruna-Concha et al. (2001) |
| Methional |  | x | boiled potato | Sulphur amino acid degradation | Petersen et al. (1999) |
| 2,4-Heptadienal | x | x | oily, mushroom | Lipid degradation | Petersen et al. (1999) |
| Decanal |  | x |  | Lipid degradation | Duckham et al. (2001) |
| 2-Nonenal | x | x | sweet, green | Lipid degradation | Petersen et al. (1999) |
| 2,4-Nonadienal | x | x | Marzipan, almond, nutty, rancid | Lipid degradation | Petersen et al. (1999) |
| 2,4-Decadienal | x | x | fatty, green, onions, chips, chip fat, hot potato, liquorice, raosty | Lipid degradation | Oruna-Concha et al. (2001) |
